# Multiple resonance type thermally activated delayed fluorescence by dibenzo [1,4] azaborine derivatives

**DOI:** 10.3389/fchem.2022.990918

**Published:** 2022-09-19

**Authors:** Jaehyun Bae, Mika Sakai, Youichi Tsuchiya, Naoki Ando, Xian-Kai Chen, Thanh Ba Nguyen, Chin-Yiu Chan, Yi-Ting Lee, Morgan Auffray, Hajime Nakanotani, Shigehiro Yamaguchi, Chihaya Adachi

**Affiliations:** ^1^ Center for Organic Photonics and Electronics Research (OPERA), Kyushu University, Fukuoka, Japan; ^2^ Department of Chemistry and Biochemistry, Kyushu University, Fukuoka, Japan; ^3^ Department of Chemistry, Graduate School of Science, Nagoya University, Nagoya, Japan; ^4^ Research Center for Materials Science (RCMS) and Integrated Research Consortium on Chemical Sciences (IRCCS), Nagoya University, Nagoya, Japan; ^5^ Institute of Transformative Bio-Molecules (WPI-ITbM), Nagoya University, Nagoya, Japan; ^6^ International Institute for Carbon Neutral Energy Research (WPI-I^2^CNER), Kyushu University, Fukuoka, Japan

**Keywords:** TADF, thermally activated delayed fluorescence, multi-resonance, azaborine, blue–violet OLED

## Abstract

We studied the photophysical and electroluminescent (EL) characteristics of a series of azaborine derivatives having a pair of boron and nitrogen aimed at the multi-resonance (MR) effect. The computational study with the STEOM-DLPNO-CCSD method clarified that the combination of a BN ring-fusion and a terminal carbazole enhanced the MR effect and spin-orbit coupling matrix element (SOCME), simultaneously. Also, we clarified that the second triplet excited state (T_2_) plays an important role in efficient MR-based thermally activated delayed fluorescence (TADF). Furthermore, we obtained a blue–violet OLED with an external EL quantum efficiency (EQE) of 9.1%, implying the presence of a pronounced nonradiative decay path from the lowest triplet excited state (T_1_).

## 1 Introduction

Recently, organic emitters providing narrowband emission attracted intense interest aimed at the display applications in organic light-emitting diodes (OLEDs) (Hall et al., 2020; Teng, et al., 2020; Ha et al., 2021; Monkman 2021) because such emitters can achieve high color purity, i.e., high color reproduction area ratio vs. the National Television System Committee (NTSC) color gamut. At the early dawn of OLED development, fluorescence materials had been widely used as an emitter (Tang and VanSlyke, 1987; Adachi et al., 1988; Dodabalapur, 1997; Hung et al., 1997; Shi and Tang., 1997). However, they provide only 25% electron-hole pair-to- photon conversion efficiency because of the spin-statistical theorem (Rothberg and Lovinger, 1996; Köhler et al., 2009). Instead, room temperature phosphorescence materials have resolved this critical problem, achieving 100% internal quantum efficiency (Baldo et al., 1998; Adachi et al., 2001). Nevertheless, the materials had several disadvantages, such as high cost, board emission spectra, and a rather long triplet lifetime, originating from the precious metal complexes and MLCT emission characters. In 2012, on the other hand, our group reported a state-of-the-art emitter based on thermally activated delayed fluorescence (TADF), realizing 100% upconversion from a lowest excited triplet (T_1_) to a lowest excited singlet (S_1_) using simple aromatic compounds (Uoyama et al., 2012). The TADF emitters can harvest both prompt emission by conventional fluorescence and delayed emission from S_1_ *via* reverse intersystem crossing (RISC) from T_1_. After this report, a wide variety of TADF materials have been developed worldwide (Nakanotani et al., 2021). However, the TADF emitters also possess the crucial problem of broad emission spectra due to the emission originating from the donor–acceptor (D-A) configuration, i.e., the formation of CT excitons to achieve a small energy splitting between S_1_ and T_1_ (Δ*E*_ST_) (Endo et al., 2009; Endo et al., 2011; Uoyama et al., 2012).

In 2016, Hatakeyama et al. (2016) reported a new type of TADF emitters, achieving narrowband emission with a full width at half maximum (FWHM) of less than 30 nm. These unique emitters were designed by using the MR effects which can localize their highest occupied and lowest unoccupied molecular orbitals (HOMO and LUMO) on the adjacent atoms constituting heterocyclic aromatic cores, achieving unique HOMO and LUMO separation without using the D-A configuration. Also, due to the rigid molecular structures, the vibrational mode of the core structures was significantly suppressed, providing narrowband emission. Furthermore, very recently, a wide variety of MR-TADF emitters were reported, providing excellent device performance in OLEDs. However, most of the reported MR-TADF emitters have a relatively large Δ*E*_ST_ compared to the well-elaborated donor–acceptor type TADF emitters, resulting in a small RISC rate (<10^5^ s^−1^) while maintaining a large radiative decay rate (>10^8^ s^−1^). Therefore, MR-TADF emitters are often used as a terminal emitter in TADF-assisted fluorescence (hyperfluorescence) devices (Nakanotani et al., 2014; Chan et al., 2021). In the recent trend, continuously researchers have been focused on obtaining stable MR-TADF emitters having the compatibility of a narrower FWHM and a large RISC rate by engineering the MR effect to achieve highly stable and high color purity OLEDs (Kondo et al., 2019; Oda et al., 2019).

**FIGURE 1:**
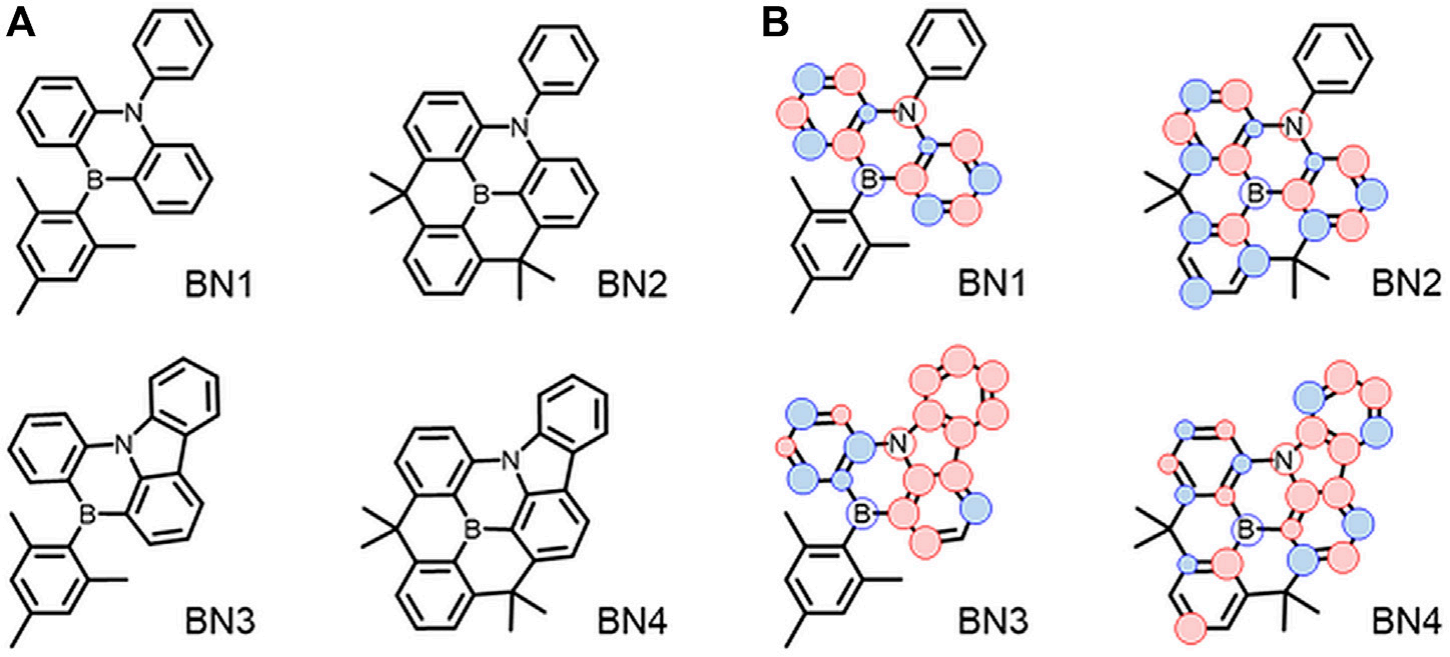
**(A)** Chemical structures of dibenzo [1,4] azaborine derivatives. **(B)** Schematic illustration of the spin density of hole (red) and electron (blue) on main backbone at S_1_ state.

Previously, we reported the synthesis of MR materials showing narrowband emission with a simple heterocyclic aromatic structure composed of a dibenzo [1,4] azaborine skeleton (**BN1**)-based core having a single couple of boron (B) and nitrogen (N) (Figure 1A) (Ando et al., 2019). This B-N core is the minimum building block to achieve HOMO-LUMO separation on heterocycles rather than the first reported MR-TADF materials of DABNA (Hatakeyama et al., 2016). Based on the scaffold of **BN1**, the ring-fused planar fashion of triarylborane and/or triarylamine to expand the π-conjugation would be expected to improve their photophysical properties (**BN2**, **BN3**, and **BN4**). In this report, we studied the detailed photophysical and OLED characteristics of these emitters. As a result, we found the original azaborine backbone has no TADF property, and the planarization of the *B*- and *N*-phenyl groups by ring-fusion provides the TADF property. Especially, the carbazole formation enhances the SOC, and the most planar **BN4** showed the best TADF characteristics. In addition, it was suggested that the major nonradiative decay of **BN4** occurs from the T_1_ state because the theoretical EQE has a considerably large difference from the experimental EQE depending on the ratio of two nonradiative decays from S_1_ and T_1_.

## 2 Results and discussion

### 2.1 Computational analysis with wave function-based methodology

First, we performed a theoretical study based on a quantum chemical calculation for **BN1-4**. Recently, Pershin et al. (2019) studied some MR materials, demonstrating the good agreement of Δ*E*_ST_ between theoretical calculation and experimental values by not using the density functional theory (DFT) method but the wave function-based method with the spin-component scaled (SCS) coupled-cluster model (CC2). While we applied these methods to **BN1-4** at first, the calculation results did not provide a good agreement with the experimental results as shown in the supplemental information (Supplementary Figure S1, Supplementary Table S1). Also, similar to the several reports of MR-TADF emitters (Hatakeyama et al., 2016; Oda et al., 2019; Lee et al., 2021), the DFT calculation with the B3LYP/6-31G(d) level (Stephen et al., 1994) provided a large difference with the experimental results for S_1_, T_1_ levels, and Δ*E*_ST_. In addition, the values of the oscillator strength (*f*) were inconsistent with the experimentally estimated ones. Next, we also used the SCS-CC2 level. While it showed better agreement with the experimental Δ*E*_ST_, it resulted in lower values when *N*-phenyl units were incorporated into the fused rings. Moreover, a considerable difference was observed in the energy levels of both singlet and triplet excited states.

**FIGURE 2:**
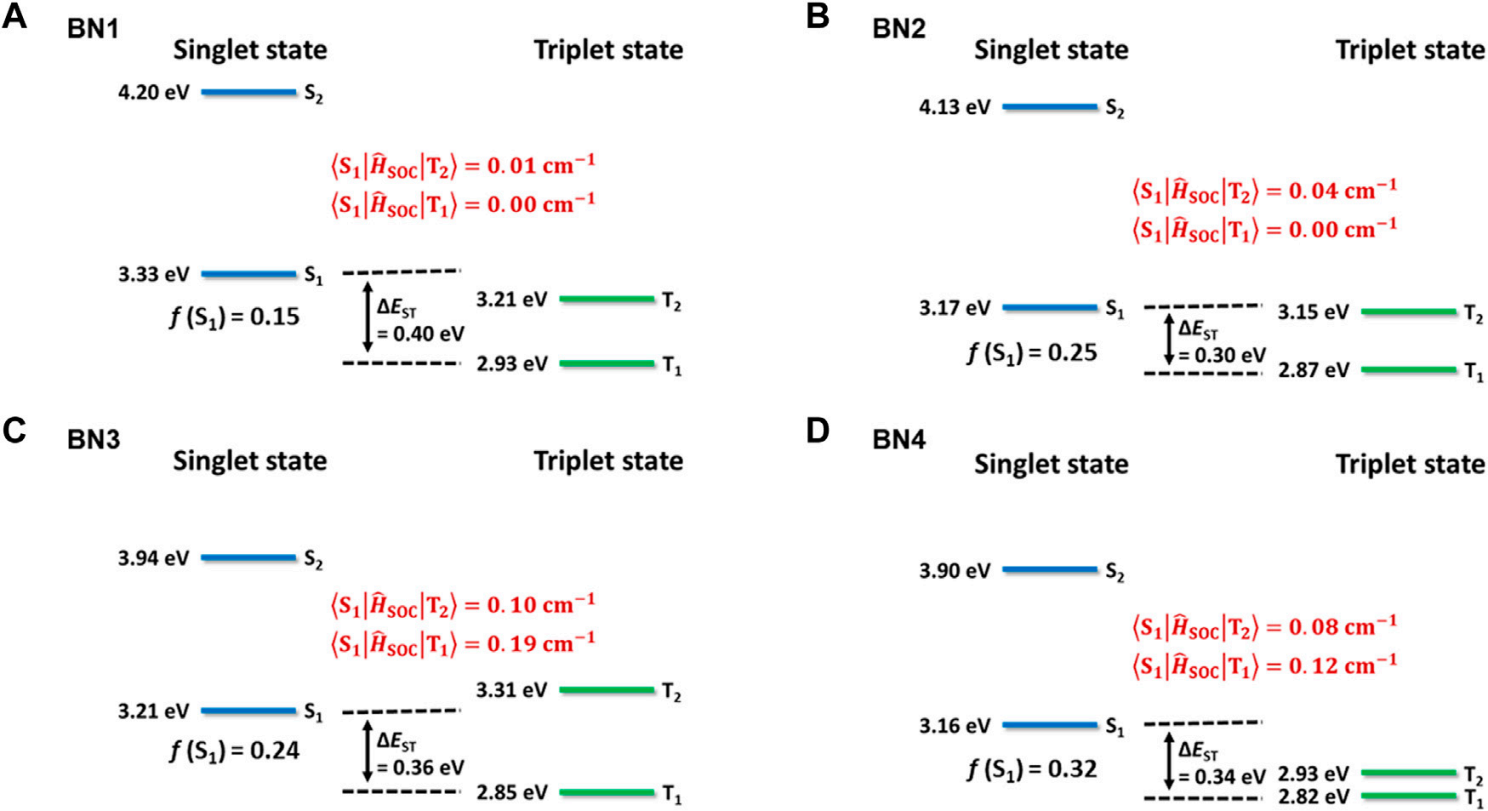
Calculated excited state energies for **(A) BN1**, **(B) BN2**, **(C) BN3**, and **(D) BN4** at the STEOM-DLPNO-CCSD level of theory. Singlet (S_1_, S_2_) and triplet (T_1_, T_2_) levels were based on the geometry optimized for each level. Oscillator strength **(*f*)** values were indicated for the S_1_–S_0_ transition at the S_1_ state geometry. Spin-orbit coupling (SOC) strengths were estimated by using initial and final state geometries.

With the comprehensive evaluation of the calculation methods, we found the Similarity Transformed Equation-Of-Motion Domain-based Local Pair Natural Orbital Coupled-Cluster Singles and Doubles (STEOM-DLPNO-CCSD) method, which is a wave function-based quantum chemistry approach based on EOM-CCSD (Stephen et al., 1994), provides the best results with the experimental values for S_1_, T_1_ levels, and Δ*E*_ST_. The calculated values of singlet, triplet levels, Δ*E*_ST_, spin-orbit coupling matrix element (SOCME), and *f* are summarized in Figure 2. The hole and electron distributions at the S_1_ state for each material are illustrated in Figure 1B. Also, the spin density difference plots for S_1_, T_1_, and T_2_ are shown in Supplementary Figure S2. Because of the higher energy of the S_2_ level than that of the S_1_, T_1_, and T_2_ levels, it is reasonable to consider only the lower three levels for the emission process in all **BN**s. Although the absolute *f* values for the **BN**s’ S_1_ levels are overestimated, the order is consistent with the π-extension trend of the ring-fused structures. The Δ*E*_ST_ values, which are also reduced by the π-extension trend, are estimated to be 0.30–0.40 eV, while these values are slightly overestimated, i.e., <0.1 eV, compared to the experimental values. Interestingly, the SOCME showed a large difference for **BN**s, which should be related to the hole and electron distributions at each state of S_1_, T_1_, and T_2_. In the case of **BN1** and **BN2**, the SOCMEs between S_1_ and T_1_ were estimated to be 0.00 cm^−1^ because of the similar hole and electron distributions, which is the forbidden transition explained by El-Sayed’s rule. Furthermore, since the SOCMEs between S_1_ and T_2_ in **BN1** and **BN2** are rather small values of 0.01 and 0.04 cm^−1^, respectively, they would show virtually no TADF activity. Instead, **BN3** and **BN4** are TADF-active. When an *N*-phenyl group is fused with the BN ring (**BN3**), the hole distribution of the S_1_ state locates mainly on the carbazole moiety, and the electron is distributed on the azaborine moiety, inducing the charge transfer (CT) nature. In the T_1_ state, both hole and electrons are distributed on the carbazole moiety, while they distribute on the azaborine moiety in the T_2_ state. These spin distribution differences among S_1_, T_1_, and T_2_ enhance the SOCME of **BN3** (0.19 and 0.10 cm^−1^ for S_1_-T_1_ and S_1_-T_2_, respectively). In the case of **BN4**, the spin distribution of T_1_ is almost as same as **BN3**, while that of the T_2_ level spreads to the whole molecule. Interestingly, the SOCME of **BN4** is as same as that with **BN3** (0.12 and 0.08 cm^−1^ for S_1_-T_1_ and S_1_-T_2_, respectively).

### 2.2 Photophysical properties of BN molecule series in solution

**FIGURE 3:**
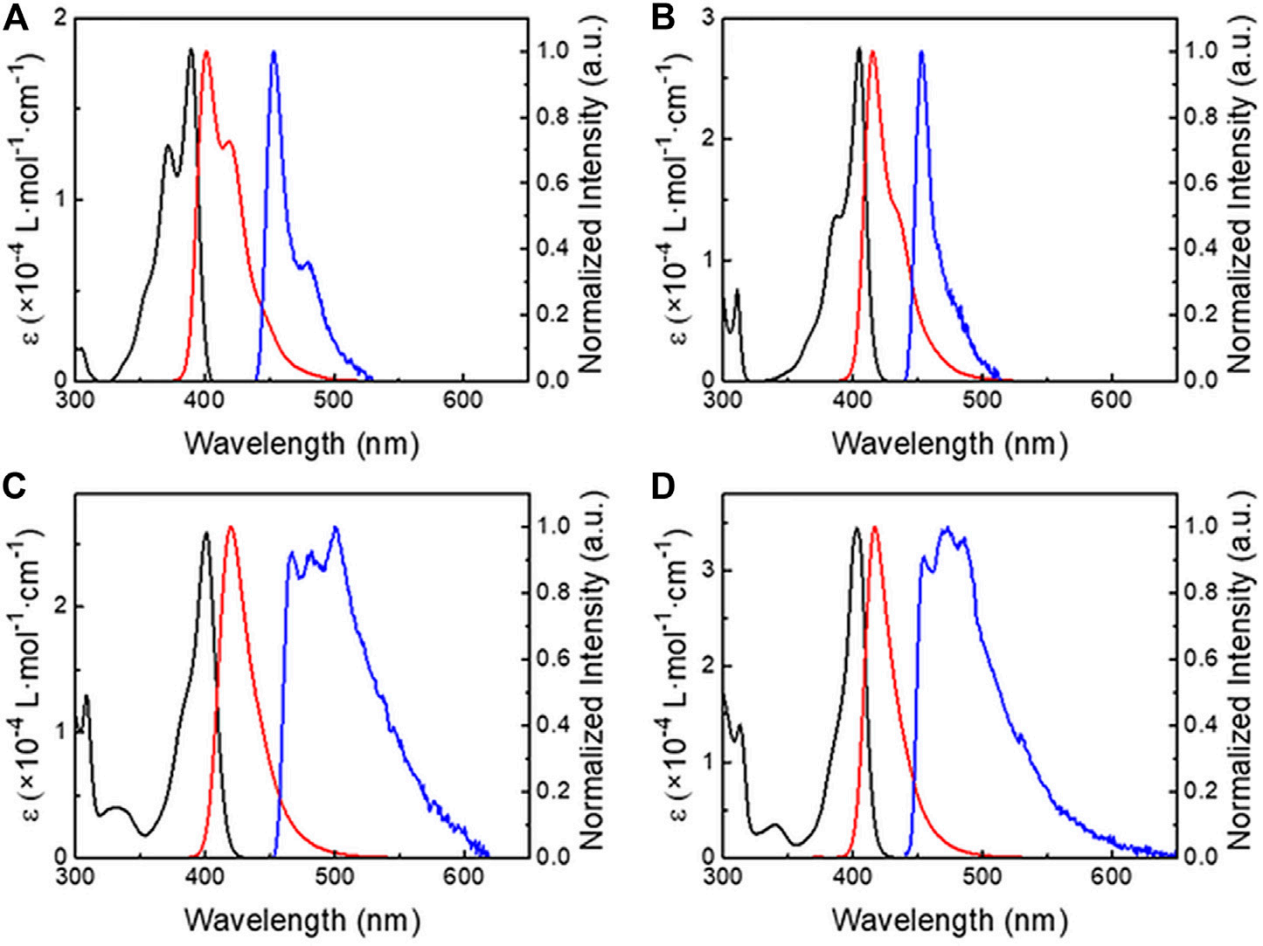
Absorption (black line), fluorescence (red line), and phosphorescence (blue line, at 77 K) spectra of **(A) BN1**, **(B) BN2**, **(C) BN3**, **(D) BN4** in toluene (1.0 × 10^–5^ mol L^−1^).

Figure 3 shows the ultraviolet–visible (UV–Vis) absorption and emission spectra of **BN1-4** in toluene (1.0 × 10^–5^ mol L^−1^). Previously, we demonstrated the estimation method of experimental *f* values for fluorescence materials using the integral of the molar absorption coefficient (*ε*) which originates from the absorption component located at the lowest energy, and we applied this method to TADF materials (Hirata et al., 2015; Tsuchiya et al., 2020; Tsuchiya et al., 2021a). Also, the transition dipole moment (*Q*) and radiative decay rate from an S_1_ state $\left(k_{r}^{S}\right)$ can be estimated with the absorption and emission spectra. Fortunately, all four compounds showed the characteristic absorption band at a longer wavelength than ca. 350 nm. Therefore, these absorption bands were separated based on the multi-component Gaussian curve fitting (Supplementary Figure S3). The intense absorption band with a peak around 400 nm can be characterized as a short-range CT transition on the polycyclic aromatic backbone, i.e., the MR nature originated from the fused BN structure. The estimated *f*, *Q*, and $k_{r}^{S}$ values were provided using the whole lowest absorption band and provided good agreement with the values of computationally estimated *f* and $k_{r}^{S}$ estimated by the transient emission decay and PLQY. The experimental *f* values were 0.110, 0.129, 0.124, and 0.138 for **BN1**, **2**, **3**, and **4**, respectively (Table 1). This trend showed good agreement with those of the calculated values: 0.15, 0.25, 0.24, and 0.32. The *B*-phenyl fused **BN2** provides a slightly larger *f* value than that of *N*-phenyl fused **BN3** both in the computational and experimental results. The spectroscopic $k_{r}^{S}$ provided ca. 20%–25% large values in all **BN**s compared with the result of transient emission decay measurement in toluene. By comparing those absorption bands, we can recognize that the condensed structures well-suppress the vibronic band in the absorption and fluorescence spectra. Particularly, **BN4** forming the carbazole is the most effective for suppression, and it showed the narrowest band emission with the FWHM of 25 nm (49 meV). The fluorescence spectra of **BN3** have no vibronic structure, but they were slightly broadened compared to those of **BN4** (29 nm). This can be ascribed to the CT-like spin distributions which were found in the theoretical calculation. The expansions of the π-conjugation by planarization also showed a slight bathochromic shift of the absorption and fluorescence spectra; each peak maxima of the absorption spectra for **BN1**, **2**, **3,** and **4** are 389, 405, 401, and 403 nm, respectively. Thus, **BN2** and **BN4** showed a slightly larger bathochromic effect than others.

**TABLE 1:** Photophysical values of **BN1**-**4** in toluene solution (1.0 × 10^–5^ mol L^−1^).

Compounds	*λ*_abs_ ^a^ (nm)	*ε* ^a^ × 10^–4^ (L mol^−1^ cm^−1^)	*λ*_flu_ ^b^ (nm)	PLQY^c^	FWHM^a^ (nm)	$S_{1}$ ^d^ (eV)	$T_{1}$ ^d^ (eV)	$E_{\mathrm{ST}}$ ^d^ (eV)	*f* ^e^	*Q* ^e^ (D)	*k*_r_^S^ ^e^ (10^8^ s^−1^)
**BN1**	389	1.83	401	0.98	36	3.20	2.81	0.40	0.110 (0.15)	1.564	1.35 (1.12)
**BN2**	405	2.75	415	0.98	28	3.08	2.80	0.28	0.129 (0.25)	1.925	1.51 (1.28)
**BN3**	401	2.59	420	0.86	29	3.09	2.72	0.37	0.124 (0.24)	2.085	1.42 (1.14)
**BN4**	403	3.44	417	0.86	25	3.09	2.78	0.30	0.138 (0.32)	2.428	1.67 (1.38)

a Absorption peak maxima of the lowest energy absorption band.

b Emission peak maxima.

c Photoluminescence quantum yield measured under inert gas conditions.

d S_1_ and T_1_ are estimated from the onset values of fluorescence and phosphorescence spectra.

e Oscillator strength.

(*f*), Transition dipole moment (*Q*) and radiative decay rate (*k*_r_^S^) are estimated from absorption and emission spectra by the reported method in literature (Tsuchiya et al., 2020). The values of *f* shown in parentheses are the computationally calculated value with the STEOM-DLPNO-CCSD, level of theory. The values of *k*_r_^S^ shown in parentheses are obtained from the emission decay rate and PLQY.

As expected in the spin distribution on the computational analysis, the *N*-phenyl planarized ones (**BN3** and **4**) showed similar broad phosphorescence spectra having vibronic structures. On the other hand, **BN1** and **2** showed narrowband phosphorescence. The fluorescence and phosphorescence in all **BN**s provided similar spectra in various solvents (Supplementary Figure S4). In other words, **BN**s have less polarity sensitivity for both S_1_ and T_1_ energies. The estimated S_1_, T_1_ energy levels, and Δ*E*_ST_ values in toluene are summarized in Table 1. In addition, the experimentally obtained $k_{r}^{S}$ also showed good agreement with the estimated $k_{r}^{S}$ from the spectral data. Unfortunately, it was very difficult to find delayed emission in toluene for all **BN**s. Even the most planar **BN4** showed a very weak delayed emission; $\Phi_{PF}:\Phi_{DF}=85\;:1$ (Supplementary Figure S5). This would be based on the small intersystem crossing (ISC) rate ( $k_{ISC}$ = (1.28 ± 1.09) × 10^7^ s^−1^) against the sum of radiative and nonradiative decay rates ( $k_{r}^{S}+k_{nr}^{S}$ = (1.44 ± 0.11) × 10^8^ s^−1^) and also the small reverse ISC rate ( $k_{RISC}$ = (9.84 ± 8.37) × 10^4^ s^−1^) against the rate of nonradiative decay from T_1_ ( $k_{nr}^{T}$ = 1.67 × 10^5^ s^−1^ in maximum). It should be noted that the maximum $k_{ISC}$ and $k_{RISC}$ correspond to the limit condition of $k_{nr}^{T}=0$ and $k_{nr}^{S}=0$ , respectively. Also, the minimum values correspond to $k_{nr}^{S}=0$ and $k_{nr}^{T}=0$ , respectively (Tsuchiya et al., 2021b).

### 2.3 Thermally activated delayed fluorescence properties from the transient decay curve in a DPEPO-doped film

**FIGURE 4:**
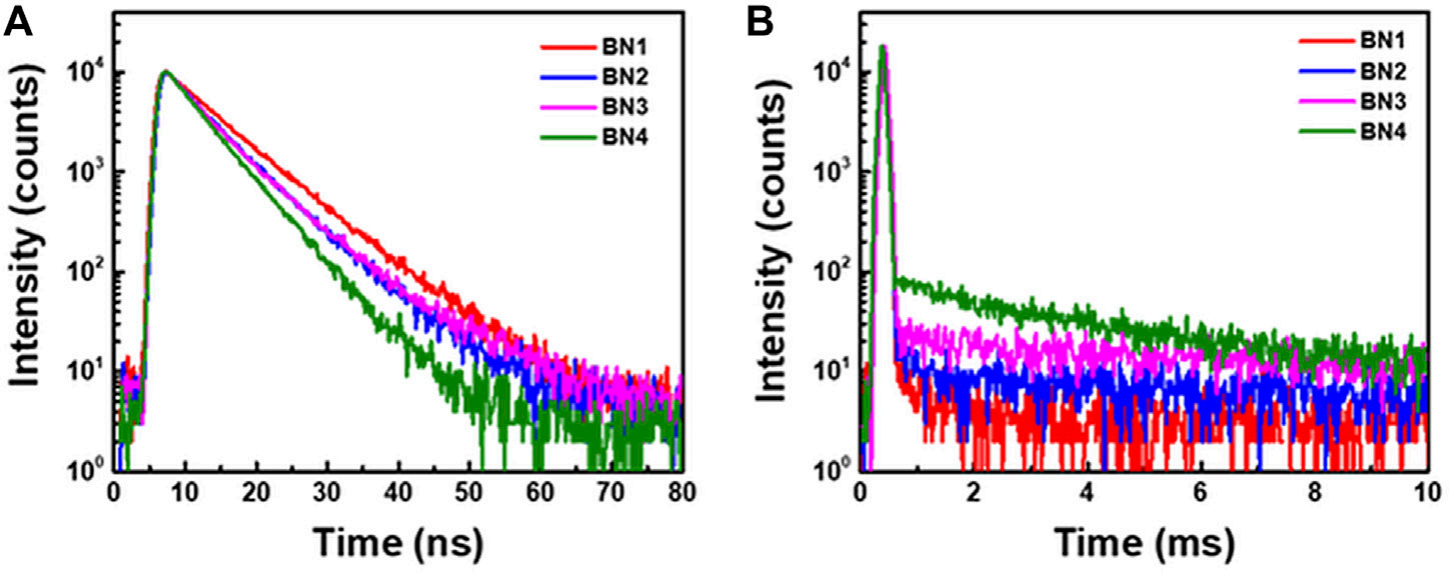
Transient PL decay curves of **(A)** prompt and **(B)** delayed emissions for **BN1**, **2**, **3**, and **4** in DPEPO films (1 wt% doped) were measured with a streak camera under the vacuum at 300 K.

Because all **BN**s would show very weak TADF property in solution, we investigated the photophysical properties of **BN**s in their solid-state films to suppress the nonradiative decays. We used bis [2-(diphenylphosphino) phenyl] ether oxide (DPEPO) as a high T_1_ host matrix to confine the high T_1_ energy of **BN**s (>2.7 eV). In addition, we fabricated **BN**-doped DPEPO films with a doping ratio of 1 wt% to inhibit the aggregation. The absorption and emission spectra of each **BN** in DPEPO showed almost the same emission spectra as those in toluene (Table 2 and Supplementary Figure S6). Thus, it was confirmed that **BN**s do not aggregate significantly in the doped films. The transient PL properties were investigated by a steak camera system with a fourth harmonic Nd:YAG laser (266 nm) under vacuum (Figure 4 and Supplementary Figure S7).

**TABLE 2:** Estimated photophysical and rate constant values of 1 wt% **BN**s in the DPEPO doped film.

Doped film	S_1_ ^a^ (eV)	T_1_ ^a^ (eV)	Δ*E*_ST_ ^a^ (eV)	τ_p_ ^b^ (ns)	τ_d_ ^b^ (ms)	*Ф*_PF_ ^c^	*Ф*_DF_ ^c^	*Ф*_PLQY_ ^d^	*k*_r_^S^ ^c^ (10^8^ s^−1^)	Max. *k*_nr_^S^ ^c^ ^,^ ^e^ (10^7^ s^−1^)	Avg. *k*_ISC_ ^c^ ^,^ ^e^ (10^7^ s^−1^)	Avg. *k*_RISC_ ^c^ ^,^ ^e^ (s^−1^)	Max. *k*_nr_^T^ ^c^ ^,^ ^e^ (s^−1^)
**BN1**	3.18	2.82	0.36	7.03	—	0.82	—	0.821	1.17	2.55	1.27 ± 1.27	—	—
**BN2**	3.06	2.78	0.28	5.70	16.24	0.70	0.06	0.762	1.24	3.86	3.25 ± 1.93	41.80 ± 25	49.60
**BN3**	3.07	2.76	0.29	5.54	13.34	0.67	0.09	0.766	1.21	3.89	4.04 ± 1.95	57.27 ± 28	55.14
**BN4**	3.07	2.83	0.24	4.96	4.19	0.70	0.10	0.801	1.40	3.49	4.38 ± 1.74	196.50 ± 78	156.51

a Estimated from onset values of fluorescence and phosphorescence spectra. Δ*E*_ST_ = S_1_ − T_1_.

b Prompt and delayed emission lifetimes were estimated from the ns and ms range transient emission decay curves, respectively.

c Values were estimated by the reported method in the literature (Tsuchiya et al., 2021b).

d Under inert gas conditions.

e Maxima values of *k*_nr_^S^ and *k*_nr_^T^ are related to the limited condition of *k*_nr_^T^ = 0, and *k*_nr_^S^ = 0, respectively. Maximum $k_{ISC}$ and minimum $k_{RISC}$ obtained from average rate constants with the range are relayed to *k*_nr_^S^ and opposite values are related to *k*_nr_^T^.

**BN**s showed a comparable radiative decay rate $\left(k_{r}^{S}=\Phi_{PF}/\tau_{p}\right)$ with a similar trend to the estimated ones from the spectroscopy, i.e., **BN1** < **BN2** ≈ **BN3** < **BN4** (Table 2). Since **BN1** showed no delayed emission even in the doped film, we conclude that the fundamental dibenzo [1,4] azaborine backbone has no TADF activity. **BN1** has a large Δ*E*_ST_ of 0.36 eV, while other BNs have relatively small Δ*E*_ST_ of less than 0.30 eV. While the orbital separation of the hole and electron shows a typical MR pattern, it holds a large orbital overlap between them. In addition, **BN1** shows a large vibronic structure in its absorption and emission spectra. These results clearly indicate that it is insufficient to achieve MR-TADF by just connecting two phenyl rings to the electron-donating and -withdrawing atoms in the azaborine backbone of **BN1**. On the other hand, the delayed emission component ratio to the total PLQY was increased by planarization; 0.079, 0.118, and 0.125 for **BN2**, **3**, and **4**, respectively. It suggests that the delayed emission is closely related to formation of the carbazole moiety, rather than planarization of the *B*-phenyl group. Since **BN2** has a smaller Δ*E*_ST_ than **BN3**, this result should be originated from the larger SOCME in **BN3**, which is in good agreement with the computational results (Figure 2). The temperature dependence of transient emission decay profiles of **BN1**-**4** (Supplementary Figure S7A) and those spectra of delayed components (1–10 ms) (Supplementary Figure S7B) clearly explained the **BN2**-**4** has TADF characteristics. **BN1** showed the only phosphorescence increase, but **BN2**-**4** showed the decrease of delayed fluorescence, which is carried out by decreasing temperature. All **BN**s emit in the fluorescence region at 50 K, but this emission would be based on the triplet–triplet annihilation. At 300 K, **BN4** showed a much shorter delayed emission lifetime ( $\tau_{d}$ = 4.2 ms) than that of **BN2** and **BN3** (16.2 and 13.3 ms, respectively). It suggests that the T_2_ state of **BN4** lies at a lower level than that of **BN3**, leading to efficient upconversion. The $k_{ISC}$ values for **BN2**, **3**, and **4** were estimated to be 1.32 × 10^7^, 2.09 × 10^7^, and 2.64 × 10^7^ s^−1^, respectively, and $k_{RISC}$ values were 66.8, 85.3, and 204.3 s^−1^, respectively (at 300 K; it can be approximated to be no phosphorescence in the delayed emission, and this approximation provides $k_{nr}^{T}=0$ ). The possible range for rate constants is summarized in Table 2 (Tsuchiya et al., 2021b). The theoretical $k_{RISC}$ can be estimated by the Marcus equation considering the density of states between T_1_ and T_2_ (Samanta et al., 2017), and the $k_{RISC}$ s for each RISC pathway was estimated (Supplementary Table S2). Considering the direct RISC process from T_1_ to S_1_, $k_{RISC}$ **BN3** is slightly larger than that of **BN4**. On the other hand, those values were inverted considering the contribution of a T_2_ state $\left(k_{RISC}^{S_{1}\leftarrow T_{2}↔T_{1}}\right)$ . When the Δ*E*_ST_ values obtained from the spectral data were introduced to the Marcus equation with the observed $k_{RISC}$ , the SOCME was estimated to be 0.068 and 0.049 cm^−1^ ( $k_{nr}^{S}=0$ ) or 0.115 and 0.075 cm^−1^ ( $k_{nr}^{T}=0$ ) for **BN3** and **BN4**, respectively. This trend agreed with the direct RISC process without considering T_2_ contribution. Furthermore, the effective SOCME was also estimated with the Marcus plot (Supplementary Figure S8) (Fukuzumi et al., 2015); the detailed estimation method is provided in Supplementary Material S1. The effective SOCMEs of the RISC process for **BN3** and **BN4** were estimated to be ca. 0.0006 and 0.004 cm^−1^, respectively. In other words, **BN4** provided a larger SOCME which is one order magnitude larger than that of **BN3** (Supplementary Table S2). Therefore, we concluded the T_n_ state enhances the RISC process in **BN4**.

### 2.4 Organic light-emitting diode device employing BN4 as an emitter

**FIGURE 5:**
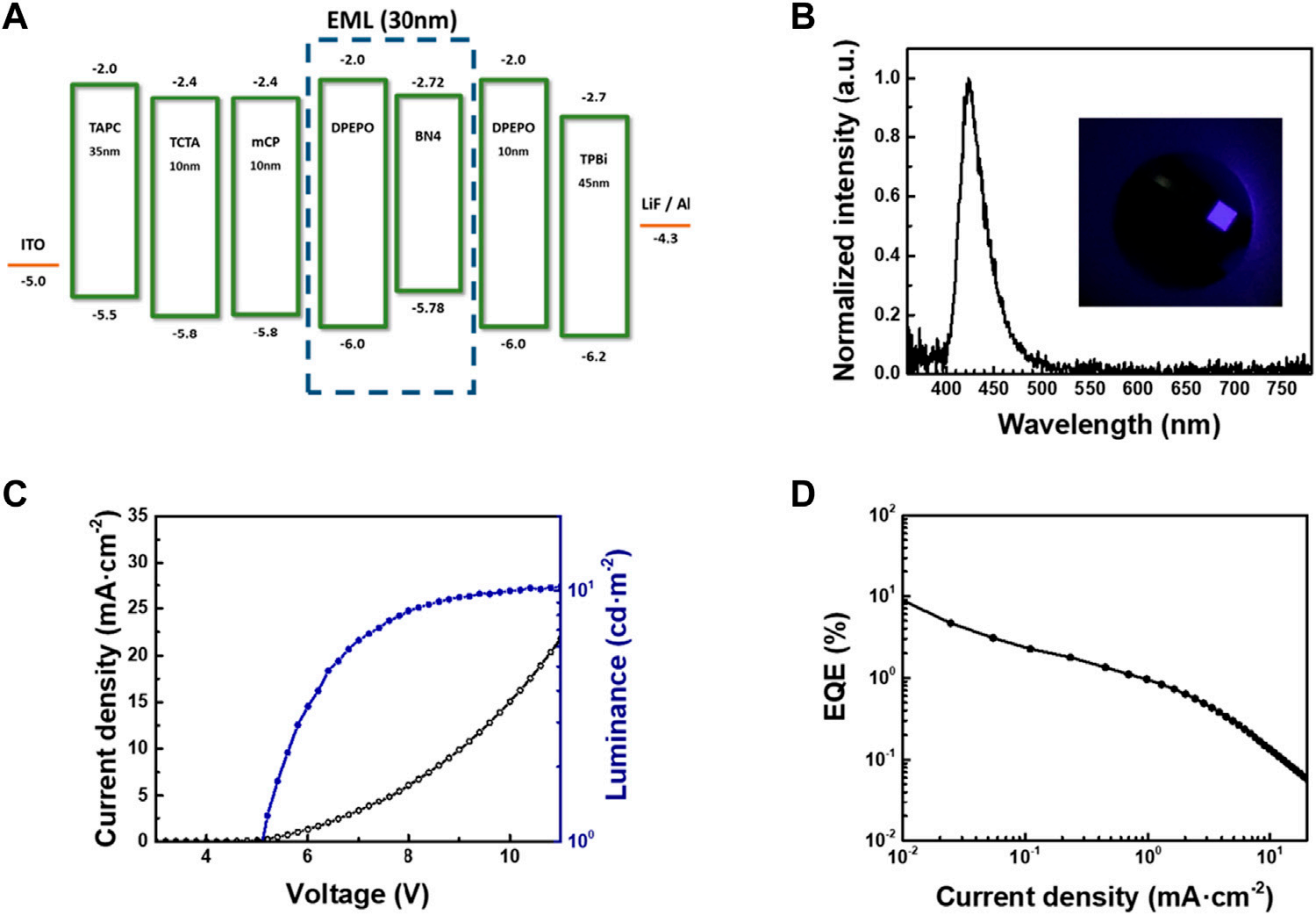
OLED device characteristics of **BN4**; **(A)** schematic device structure and energy level alignment, **(B)** normalized EL spectra, **(C)** J-V-L profile, and **(D)** EQE-J curves.

Finally, we fabricated an OLED with **BN4** as an emitter (Figure 5) with the device structure of indium tin oxide (ITO)/1,1-bis [(di-4-tolylamino) phenyl]cyclohexane (TAPC, 35 nm)/4,4′,4″-tris (carbazol-9-yl)-triphenylamine (TCTA, 10 nm)/1,3-bis (9-carbazol-9-yl) benzene (mCP, 10 nm)/DPEPO doped with 1 wt% of **BN4** (30 nm)/DPEPO (10 nm)/2,2′,2"-(1,3,5-benzinetriyl) -tris (1-phenyl-1-*H*-benzimidazole) (TPBi, 45 nm)/LiF (0.8 nm)/Al (100 nm). The electroluminescence spectrum showed good agreement with the PL spectrum of **BN4** in a DPEPO host (*λ*_EL,max_ = 423 nm, FWHM = 31 nm). The CIE coordinates were (0.17, 0.04). The maximum EQE ( $EQE_{max}$ ) was 9.1%. Here, $EQE_{max}$ can be theoretically estimated with Eq. 1.

$$\begin{array}{c} EQE_{max}=\eta_{int}\eta_{out}\eta_{CB} \end{array},$$ (1)

where $\eta_{int}$ is an internal quantum efficiency, $\eta_{out}$ is an outcoupling efficiency, and $\eta_{CB}$ is a charge balance factor (ideally, $\eta_{CB}=1$ ). $\eta_{int}$ can be explained by the emission efficiency from the generated S_1_ and T_1_ excitons, with the exciton generation efficiency of 1:3 for S_1_:T_1_. In the case of fluorescence and phosphorescence devices, $\eta_{int}$ can be explained with effective exciton generation efficiency ( $\eta_{exc}$ ) and PLQY ( $\Phi_{PLQY}$ ) as given by Eq. 2.

$$\begin{array}{c} \eta_{int}=\eta_{exc}\Phi_{PLQY} \end{array}$$, (2)

Recently, this equation has been widely applied to TADF OLEDs since it is reasonable to apply it for highly efficient emitters, i.e., $k_{nr}^{S}=0$ or $k_{nr}^{T}=0$ (Masui et al., 2013; Dias et al., 2017). However, some TADF molecules possess the nonradiative rates of $k_{nr}^{S}\neq 0$ or $k_{nr}^{T}\neq 0$ , and we corrected the theoretical equation for $\eta_{int}$ as Eq. 3 which had been already reported (Endo et al., 2011; Hirata et al., 2015); the derivation is provided in Supplementary Material S1.

$$\begin{array}{c} \eta_{int}=\frac{1}{4}\left(\Phi_{PLQY}+3\frac{\Phi_{DF}}{\Phi_{ISC}}\right) \end{array},$$ (3)

where $\Phi_{ISC}$ is $k_{ISC}/\left(k_{r}^{S}+k_{nr}^{S}+k_{ISC}\right)$ . The term of $\Phi_{DF}/\Phi_{ISC}$ becomes $\Phi_{PLQY}$ when $k_{nr}^{T}$ is accurately 0, and it becomes the same as Eq. 2. It should be noted that $\eta_{exc}=1$ should be considered a maximum value for TADF, meaning that Eq. 2 should not be used when $\Phi_{PLQY}$ is not unity. In fact, $k_{nr}^{T}=0$ is just a mathematically reasonable value when we can approximate no phosphorescence in emission (Tsuchiya et al., 2021a). When there is the nonradiative path from the T_1_ state, it affects 75% of generated triplet excitons by current excitation at first. The ratio of the nonradiative path from a T_1_ state in PL is given by $\Phi_{ISC}\Phi_{nr}^{T}+\overset{\infty }{\underset{n=1}{\sum }}{\left(\Phi_{ISC}\Phi_{RISC}\right)}^{n}\Phi_{nr}^{T}$ , while that in EL is given by $\left(0.25\Phi_{ISC}+0.75\right)\Phi_{nr}^{T}+\overset{\infty }{\underset{n=1}{\sum }}{\left(\Phi_{ISC}\Phi_{RISC}\right)}^{n}\Phi_{nr}^{T}$ where $\Phi_{nr}^{T}=k_{nr}^{T}/\left(k_{r}^{T}+k_{nr}^{T}+k_{RISC}\right)$ . This difference makes $\eta_{int}\neq \Phi_{PLQY}$ for TADF when $k_{nr}^{T}$ is not 0 accurately. When $\eta_{out}$ = 0.2 (which is often employed for the case of the random orientation of molecular alignment of emitters in devices) was employed, the theoretical $EQE_{max}$ for the **BN4** device is calculated as 16.0% and 9.2% for the limit condition of $k_{nr}^{T}=0$ and $k_{nr}^{S}=0$ , respectively. Therefore, it can be concluded that the most of nonradiative decay of **BN4** occurs from the T_1_ state in DPEPO.

## 3 Conclusion

In this study, we focused on the minimum structural component of an azaborine backbone structure, which is necessary to provide MR-TADF characteristics. By planarization with the ring fusion of *B*- or *N*-phenyls, the molecular structure became more rigid, demonstrating clear MR characteristics with suppressing vibronic structures in their spectra. In particular, unlike *B*-phenyl planarization, which reduces FWHM and Δ*E*_ST_ by simply extending π-conjugation, it was confirmed that the vibronic structures of emission were suppressed and molecular SOCME was improved through planarization of the *N*-phenyl moiety. Furthermore, we confirmed that the ring fusion of *B*- or *N*-phenyls showed a synergistic effect of them. The TADF nature was mainly dependent on the SOCME because **BN**s showed an appreciably large Δ*E*_ST_ (0.24–0.36 eV). Carbazole formation enhanced the SOC effectively, and further planarization reduced the T_2_ state energy and promoted the RISC process. Finally, we fabricated the OLED having blue–violet emission (*λ*_max_, 423 nm) with EQE = 9.1%, demonstrating good agreement with the maximum theoretical value of EQE = 9.2% when the nonradiative decay from the T_1_ state is considered. Considering the high PLQY of over 80% in the **BN4** film, it clearly indicates that suppressing the nonradiative process from T_1_ is mandatory for efficient MR-TADF OLED in the blue–violet region.

## 4 Materials and methods

### 4.1 Chemicals and instruments

All solvents were used as purchased from the Tokyo Chemical Industry (Tokyo, Japan) or Fuji Film-Wako Chemicals (Tokyo, Japan). All synthesis procedures for **BN1**-**4** were provided in the previous report (Ando et al., 2019). Absorption spectra of the samples were measured using an ultraviolet–visible,-near-infrared spectrometer (Lambda 950-PKA, Perkin-Elmer, MA, United States). Fluorescence and phosphorescence spectra were measured using a spectrofluorometer (FP-8600, JASCO International, Japan). The photoluminescence quantum yield (PLQY) was measured using a PLQY measurement system (Quantaurus-QY, Hamamatsu Photonics, Hamamatsu, Japan). The transient PL decay characteristics of **BN4** in toluene were recorded by a dynamic range streak camera system (C10910-01, Hamamatsu Photonics, Hamamatsu, Japan) with a third harmonic YAG laser (355 nm, 10 Hz, PL-2250, EKSPLA, Lithuania) as an excitation source. The transient PL emission and PL decay of **BN1**, **2**, **3**, and **4** doped in a DPEPO host film were recorded under vacuum with a streak camera (C4334, Hamamatsu Photonics, Hamamatsu, Japan) with a third harmonic YAG laser (266 nm, 10 Hz, LS-2132UTF, LOTIS TII, Belarus) as an excitation source.

### 4.2 Theoretical calculations

The **BN**s were analyzed by DFT calculation with the B3LYP/6-31 + g* (Lee et al., 1988; Beck, 1993) level of theory on Gaussian 16 (Frisch et al., 2016), SCS-CC2 level of theory on TURBOMOLE 7.1.1 (2016), and STEOM-DLPNO-CCSD level of theory on ORCA 4.2.1 software (Neese et al., 2020).

### 4.3 Photophysical measurement

The photophysical properties of **BN**s were measured in toluene solution (1.0 × 10^–5^ mol L^−1^), which was saturated with inert gas. Also, thermally evaporated films composed of 1 wt% emitter in a DPEPO host were measured under the vacuum condition. The transient PL decay profiles of streak camera measurement were analyzed with the ex-Gauss fitting using Eqs 4, 5; the detailed explanation is written in Supplementary Material S1.

$$\begin{array}{c} I\left(t\right)=\sum_{n=1}^{n}\left\{R_{n}\left[\overset{m}{\underset{m=1}{\sum }}I_{m}\left(t_{n},A_{\text{m}},k_{m},\mu_{n},\sigma_{n}\right)\right]\right\} \end{array},$$ (4)

,$$\begin{array}{c} I_{m}\left(t_{n},A_{m},k_{m},\mu_{n},\sigma_{n}\right)=\frac{A_{m}}{2}\exp\left[\frac{k_{m}}{2}\left(2\mu_{n}+k_{m}\sigma_{n}^{2}\right)\right]\text{erfc}\left(\frac{\mu_{n}+k_{m}\sigma_{n}^{2}-t_{n}}{\sqrt{2}\sigma_{n}}\right)\exp\left(-k_{m}t_{n}\right) \end{array}.$$ (5)

In this time, *n* = 3 and m = 2 were employed to explain three G curves, i.e., probability density functions, as an instrument-related function (IRF) and bi-exponential curve as an emission decay. $R_{n}$ is the scaling factor for each Gaussian curve to explain IRF and $t_{n}$ is $t-\mu_{n}+\mu_{1}$ . $A_{m}$ and $k_{m}$ are corresponding to the pre-exponential factor and the decay rate for the bi-exponential curve as an analytical target, respectively. $\mu_{n}$ and $\sigma_{n}^{2}$ are the terms of the mean and the variance of each Gauss curve, respectively.

## Data Availability

The original contributions presented in the study are included in the article/Supplementary Material; further inquiries can be directed to the corresponding authors.

## References

[B1] Adachi C. Baldo M. A. Thompson M. E. Forrest S. R. (2001). Nearly 100% internal phosphorescence efficiency in an organic light emitting device. J. Appl. Phys. 90, 5048–5051. 10.1063/1.1409582

[B2] Adachi C. Tokito S. Tsutsui T. Saito S. (1988). Electroluminescence in organic films with three-layer structure. Jpn. J. Appl. Phys. 27, L269–L271. 10.1143/JJAP.27.L269

[B3] Ando M. Sakai M. Ando N. Hirai M. Yamaguchi S. (2019). Planarized: B, N -phenylated dibenzoazaborine with a carbazole substructure: Electronic impact of the structural constraint. Org. Biomol. Chem. 17, 5500–5504. 10.1039/c9ob00934e 31112202

[B4] Baldo M. A. O’Brien D. F. You Y. Shoustikov A. Sibley S. Thompson M. E. (1998). Highly efficient phosphorescent emission from organic electroluminescent devices. Nature 395, 151–154. 10.1038/25954

[B5] Beck A. D. (1993). Density-functional thermochemistry. III. The role of exact exchange. J. Chem. Phys. 98, 5648–5652. 10.1063/1.464913

[B6] Chan C. Y. Tanaka M. Lee Y. T. Wong Y. W. Nakanotani H. Hatakeyama T. (2021). Stable pure-blue hyperfluorescence organic light-emitting diodes with high-efficiency and narrow emission. Nat. Photonics 15, 203–207. 10.1038/s41566-020-00745-z

[B7] Dias F. B. Penfold T. J. Monkman A. P. (2017). Photophysics of thermally activated delayed fluorescence molecules. Methods Appl. Fluoresc. 5, 012001. 10.1088/2050-6120/aa537e 28276340

[B8] Dodabalapur A. (1997). Organic light emitting diodes. Solid State Commun. 102, 259–267. 10.1016/S0038-1098(96)00714-4

[B9] Endo A. Ogasawara M. Takahashi A. Yokoyama D. Kato Y. Adachi C. (2009). Thermally activated delayed fluorescence from Sn4+-porphyrin complexes and their application to organic light-emitting diodes -A novel mechanism for electroluminescence. Adv. Mat. 21, 4802–4806. 10.1002/adma.200900983 21049498

[B10] Endo A. Sato K. Yoshimura K. Kai T. Kawada A. Miyazaki H. (2011). Efficient up-conversion of triplet excitons into a singlet state and its application for organic light emitting diodes. Appl. Phys. Lett. 98, 083302. 10.1063/1.3558906

[B11] Frisch M. J. Trucks G. W. Schlegel H. B. Scuseria G. E. Robb M. A. Cheeseman J. R. (2016). Gaussian 16, revision A.03. Wallingford CT: Gaussian Inc.

[B12] Fukuzumi S. Itoh A. Ohkubo K. Suenobu T. (2015). Size-selective incorporation of donor–acceptor linked dyad cations into zeolite Y and long-lived charge separation. RSC Adv. 5, 45582–45585. 10.1039/c5ra06165b

[B13] Ha J. M. Hur S. H. Pathak A. Jeong J. E. Woo H. Y. (2021). Recent advances in organic luminescent materials with narrowband emission. NPG Asia Mat. 13, 53. 10.1038/s41427-021-00318-8

[B14] Hall D. Suresh S. M. dos Santos P. L. Duda E. Bagnich S. Pershin A. (2020). Improving processability and efficiency of resonant TADF emitters: A design strategy. Adv. Opt. Mat. 8, 1901627. 10.1002/adom.201901627

[B15] Hatakeyama T. Shiren K. Nakajima K. Nomura S. Nakatsuka S. Kinoshita K. (2016). Ultrapure blue thermally activated delayed fluorescence molecules: Efficient HOMO-LUMO separation by the multiple resonance effect. Adv. Mat. 28, 2777–2781. 10.1002/adma.201505491 26865384

[B16] Hirata S. Sakai Y. Masui K. Tanaka H. Lee S. Y. Nomura H. (2015). Highly efficient blue electroluminescence based on thermally activated delayed fluorescence. Nat. Mat. 14, 330–336. 10.1038/nmat4154 25485987

[B17] Hung L. S. Tang C. W. Mason M. G. (1997). Enhanced electron injection in organic electroluminescence devices using an Al/LiF electrode. Appl. Phys. Lett. 70, 152–154. 10.1063/1.118344

[B18] Kondo Y. Yoshiura K. Kitera S. Nishi H. Oda S. Gotoh H. (2019). Narrowband deep-blue organic light-emitting diode featuring an organoboron-based emitter. Nat. Photonics 13, 678–682. 10.1038/s41566-019-0476-5

[B19] Lee C. Yang W. Parr R. G. (1988). Development of the Colle-Salvetti correlation-energy formula into a functional of the electron density. Phys. Rev. B 37, 785–789. 10.1103/PhysRevB.37.785 9944570

[B20] Lee Y. T. Chan C. Y. Tanaka M. Mamada M. Balijapalli U. Tsuchiya Y. (2021). Investigating HOMO energy levels of terminal emitters for realizing high-brightness and stable TADF-assisted fluorescence organic light-emitting diodes. Adv. Electron. Mat. 7, 2001090–2001099. 10.1002/aelm.202001090

[B21] Masui K. Nakanotani H. Adachi C. (2013). Analysis of exciton annihilation in high-efficiency sky-blue organic light-emitting diodes with thermally activated delayed fluorescence. Org. Electron. 14, 2721–2726. 10.1016/j.orgel.2013.07.010

[B22] Monkman A. (2021). Why do we still need a stable long lifetime deep blue OLED emitter? ACS Appl. Mat. Interfaces 14, 20463–20467. 10.1021/acsami.1c09189 PMC9100479 34232627

[B23] Nakanotani H. Higuchi T. Furukawa T. Masui K. Morimoto K. Numata M. (2014). High-efficiency organic light-emitting diodes with fluorescent emitters. Nat. Commun. 5, 4016–4017. 10.1038/ncomms5016 24874292

[B24] Nakanotani H. Tsuchiya Y. Adachi C. (2021). Thermally-activated delayed fluorescence for light-emitting devices. Chem. Lett. 50, 938–948. 10.1246/cl.200915

[B25] Neese F. Wennmohs F. Becker U. Riplinger C. (2020). The ORCA quantum chemistry Program package. J. Chem. Phys. 152, 224108. 10.1063/5.0004608 32534543

[B26] Oda S. Kawakami B. Kawasumi R. Okita R. Hatakeyama T. (2019). Multiple resonance effect-induced sky-blue thermally activated delayed fluorescence with a narrow emission band. Org. Lett. 21, 9311–9314. 10.1021/acs.orglett.9b03342 31613109

[B27] Pershin A. Hall D. Lemaur V. Sancho-Garcia J. C. Muccioli L. Zysman-Colman E. (2019). Highly emissive excitons with reduced exchange energy in thermally activated delayed fluorescent molecules. Nat. Commun. 10, 597–7. 10.1038/s41467-019-08495-5 30723203 PMC6363735

[B28] Rothberg L. J. Lovinger A. J. (1996). Status of and prospects for organic electroluminescence. J. Mat. Res. 11, 3174–3187. 10.1557/JMR.1996.0403

[B29] Samanta P. K. Kim D. Coropceanu V. Brédas J.-L. (2017). Up-conversion intersystem crossing rates in organic emitters for thermally activated delayed fluorescence: Impact of the nature of singlet vs triplet excited states. J. Am. Chem. Soc. 139, 4042–4051. 10.1021/jacs.6b12124 28244314

[B30] Shi J. Tang C. W. (1997). Doped organic electroluminescent devices with improved stability. Appl. Phys. Lett. 70, 1665–1667. 10.1063/1.118664

[B31] Stephen P. J. Devlin F. J. Chabalowski C. F. Frisch M. J. (1994). *Ab initio* calculation of vibrational absorption and circular dichroism spectra using density functional force fields. J. Phys. Chem. 98, 11623–11627. 10.1021/j100096a001

[B32] Tang C. W. VanSlyke S. A. (1987). Organic electroluminescent diodes. Appl. Phys. Lett. 51, 913–915. 10.1063/1.98799

[B33] Teng J. M. Wang Y. F. Chen C. F. (2020). Recent progress of narrowband TADF emitters and their applications in OLEDs. J. Mat. Chem. C 8, 11340–11353. 10.1039/d0tc02682d

[B34] Tsuchiya Y. Diesing S. Bencheikh F. Wada Y. dos Santos P. L. Kaji H. (2021a). Exact solution of kinetic analysis for thermally activated delayed fluorescence materials. J. Phys. Chem. A 125, 8074–8089. 10.1021/acs.jpca.1c04056 34473511

[B35] Tsuchiya Y. Ishikawa Y. Lee S. H. Chen X. K. Brédas J. L. Nakanotani H. (2021b). Thermally activated delayed fluorescence properties of trioxoazatriangulene derivatives modified with electron donating groups. Adv. Opt. Mat. 9, 2002174–2002178. 10.1002/adom.202002174

[B36] Tsuchiya Y. Tsuji K. Inada K. Bencheikh F. Geng Y. Kwak H. S. (2020). Molecular design based on donor-weak donor scaffold for blue thermally-activated delayed fluorescence designed by combinatorial DFT calculations. Front. Chem. 8, 403–411. 10.3389/fchem.2020.00403 32435635 PMC7218164

[B37] Uoyama H. Goushi K. Shizu K. Nomura H. Adachi C. (2012). Highly efficient organic light-emitting diodes from delayed fluorescence. Nature 492, 234–238. 10.1038/nature11687 23235877

