# Supplementary material for: Multiple resonance type thermally activated delayed fluorescence by dibenzo [1,4] azaborine derivatives
Source: Front Chem. 2022 Sep 19;10:990918. doi: 10.3389/fchem.2022.990918 (PMC9527295; doi:10.3389/fchem.2022.990918)
Supplement: Supplementary file 1 [file DataSheet1.docx]

Supplementary Material

# Fitting method for transient PL decay curves measured by streak camera.

The transient PL decay curve measured by a streak camera system includes the considerable instrument-related function (IRF) related to pumping laser, slit width in front of the photo-cathode, and emission distribution on phosphor plate after the microchannel plate, etc. However, the data would be analyzed with the direct multi-exponential curve fitting in general. Because the transient PL data provided the very weak delayed emission component in this study, it required a more precise analysis. Therefore, all data was analyzed with the unusual fitting method considering the IRF.

When IRF data is provided, we would be able to employ the convolution fitting provided with some software. However, there are many cases in which the IRF data cannot be used. However, the IRF can be explained by the sum of several gauss curves in many cases. Therefore, we employed an ex-gauss function for fitting the PL decay curve. The ex-gauss function is a convoluted function as explained by Eq. S1.1-S1.3.

$$\begin{aligned} f\left( x; \mu, \sigma, \lambda\right)=f\left( x \right)\bigotimes g\left( x \right)=\frac{\lambda}{2}\exp\left[ \frac{\lambda}{2}\left( 2\mu+\lambda\sigma^{2}-2x \right) \right]\mathrm{erfc} \left( \frac{\mu+\lambda\sigma^{2}-x}{\sqrt{2}\sigma} \right)\#\left( S1.1 \right) \end{aligned}$$

$$\begin{aligned} f\left( x \right)=\left\{ \begin{aligned} &\lambda\exp\left( -\lambda x \right), &\left( x\geq0 \right) \\ &0, &\left( x<0 \right) \end{aligned} \right.\#\left( S1.2 \right) \end{aligned}$$

$$\begin{aligned} g\left( x \right)=\frac{1}{\sqrt{2\pi}\sigma}\exp\left[ -\frac{\left( x-\mu\right)^{2}}{2\sigma^{2}} \right]\#\left( S1.3 \right) \end{aligned}$$

where $\mathrm{erfc} \left( x \right)$ is a complementary error function for the gauss function defined as Eq. S1.4.

$$\begin{aligned} \mathrm{erfc} \left( x \right)\equiv\frac{2}{\sqrt{\pi}}\int_{x}^{\infty} \exp\left( -x^{2} \right)dx\#\left( S1.4 \right) \end{aligned}$$

For the single exponential decay containing simple IRF explained with a single gauss curve, e.g., fluorescence emission decay, equation S1 can be rewritten as Eq. S1.5-S1.7.

$$\begin{aligned} I_{FL}\left( t \right)=f\left( t \right)\bigotimes g\left( t \right)=\frac{A}{2}\exp\left[ \frac{k_{FL}}{2}\left( 2\mu+k_{FL}\sigma^{2} \right) \right]\mathrm{erfc} \left( \frac{\mu+k_{FL}\sigma^{2}-t}{\sqrt{2}\sigma} \right)\exp\left( -k_{FL}t \right)\#\left( S1.5 \right) \end{aligned}$$

$$\begin{aligned} f\left( t \right)=\left\{ \begin{aligned} &A\exp\left( -k_{FL}t \right), &\left( t\geq0 \right) \\ &0, &\left( t<0 \right) \end{aligned} \right.\#\left( S1.6 \right) \end{aligned}$$

$$\begin{aligned} g\left( t \right)=\frac{1}{\sqrt{2\pi}\sigma}\exp\left[ -\frac{\left( t-\mu\right)^{2}}{2\sigma^{2}} \right]\#\left( S1.7 \right) \end{aligned}$$

The expanded equation for multi-exponential decay with IRF with multi-gauss function can be written by Eq. S1.8-S1.9. The formula can be written as multiple ex-gauss functions for the multiple exponential decays.

$$\begin{aligned} I\left( t \right)=\sum_{n=1}^{n} \left\{ R_{n}\left[ \sum_{m=1}^{m} I_{m}\left( t_{n},A_{m},k_{m},\mu_{n},\sigma_{n} \right) \right] \right\}\#\left( S1.8 \right) \end{aligned}$$

$$\begin{aligned} I_{m}\left( t_{n},A_{m},k_{m},\mu_{n},\sigma_{n} \right)=\frac{A_{m}}{2}\exp\left[ \frac{k_{m}}{2}\left( 2\mu_{n}+k_{m}{\sigma_{n}}^{2} \right) \right]\mathrm{erfc} \left( \frac{\mu_{n}+k_{m}{\sigma_{n}}^{2}-t_{n}}{\sqrt{2}\sigma_{n}} \right)\exp\left( -k_{m}t_{n} \right)\#\left( S1.9 \right) \end{aligned}$$

$R_{n}$ explained the relative intensity for each ex-gauss function and $t_{n}$ is $t-\mu_{n}+\mu_{1}$. The deconvoluted exponential curve can be written by Eq. S1.10.

$$\begin{aligned} I_{dec}\left( t \right)=\sum_{n=1}^{n} R_{n}\times\sum_{n=1}^{n} \left[ A_{n}\exp\left( -k_{n}t \right) \right]\#\left( S1.10 \right) \end{aligned}$$

The actual formula of Eq. S8 can be written as multiple ex-gauss functions for multiple exponential decays. To analyze the TADF on the three-state system, for example, the fitting curve for bi-exponential decay with three gauss curves as IRF can be written by Eq. S1.11-S1.13.

$$\begin{aligned} I\left( t \right)=&R_{1}\left[ I_{p}\left( t_{1},A_{p},k_{p},\mu_{1},\sigma_{1} \right)+I_{d}\left( t_{1},A_{d},k_{d},\mu_{1},\sigma_{1} \right) \right] \\ &+R_{2}\left[ I_{p}\left( t_{2},A_{p},k_{p},\mu_{2},\sigma_{2} \right)+I_{d}\left( t_{2},A_{d},k_{d},\mu_{2},\sigma_{2} \right) \right] \\ &+R_{3}\left[ I_{p}\left( t_{3},A_{p},k_{p},\mu_{3},\sigma_{3} \right)+I_{d}\left( t_{3},A_{d},k_{d},\mu_{3},\sigma_{3} \right) \right]\#\left( S1.11 \right) \end{aligned}$$

$$\begin{aligned} I_{p}\left( t_{n},A_{p},k_{p},\mu_{n},\sigma_{n} \right)=\frac{A_{p}}{2}\exp\left[ \frac{k_{p}}{2}\left( 2\mu_{n}+k_{p}{\sigma_{n}}^{2} \right) \right]\mathrm{erfc} \left( \frac{\mu_{n}+k_{p}{\sigma_{n}}^{2}-t_{n}}{\sqrt{2}\sigma_{n}} \right)\exp\left( -k_{p}t_{n} \right)\#\left( S1.12 \right) \end{aligned}$$

$$\begin{aligned} I_{d}\left( t_{n},A_{d},k_{d},\mu_{n},\sigma_{n} \right)=\frac{A_{d}}{2}\exp\left[ \frac{k_{d}}{2}\left( 2\mu_{n}+k_{d}{\sigma_{n}}^{2} \right) \right]\mathrm{erfc} \left( \frac{\mu_{n}+k_{d}{\sigma_{n}}^{2}-t_{n}}{\sqrt{2}\sigma_{n}} \right)\exp\left( -k_{d}t_{n} \right)\#\left( S1.13 \right) \end{aligned}$$

where $t_{1}$, $t_{2}$, and $t_{3}$ are $t$, $t-\mu_{2}+\mu_{1}$, and $t-\mu_{3}+\mu_{1}$, respectively. The corresponding deconvoluted exponential curve can be written by Eq. S1.14.

$$\begin{aligned} I_{dec}\left( t \right)=\left( R_{1}+R_{2}+R_{3} \right)\left[ A_{p}\exp\left( -k_{p}t \right)+A_{d}\exp\left( -k_{d}t \right) \right], &\left( t\geq0 \right)\#\left( S1.14 \right) \end{aligned}$$

In this study, the PL decay curves were analyzed by using three ex-gauss curves for bi-exponential decay with a baseline ($I\left( t \right)+baseline$).

# Estimation of experimental reorganization energy estimation (*λ*) and SOCME.

Basically, the ISC and RISC process can be explained as an electron transfer reaction between S_1_ and T_1_ orbitals therefore, those are often explained by using the basic equation of Marcus theory with the equation of Eq. S2.1-S2.2 (Marcus 1993).

$$\begin{aligned} k_{ET}=\frac{2\pi}{\hbar}\left| H_{\mathrm{if}} \right|^{2}\frac{1}{\sqrt{4\pi\lambda k_{B}T}}\exp\left[ -\frac{\Delta G^{\ddagger}}{k_{B}T} \right]\#\left( S2.1 \right) \end{aligned}$$

$$\begin{aligned} \Delta G^{\ddagger}=\frac{\left( \lambda+\Delta G^{o} \right)^{2}}{4\lambda}\#\left( S2.2 \right) \end{aligned}$$

where $k_{ET}$ is an electron transfer rate, $\hbar$ is the Dirac's constant, $H_{\mathrm{if}}$ is an electronic coupling between the initial and final states, $\Delta G^{\ddagger}$ is the transition state energy, $\lambda$ is the reorganization energy, $k_{B}$ is the Boltzmann constant, $T$ is the temperature, and $\Delta G^{o}$ is the total Gibbs free energy change between the initial and final states for the electron transfer reaction. To apply RISC process, the equation can be written as Eq. S2.3 (Samanta et at., 2017); the equation was shown only for RISC process in here.

$$\begin{aligned} k_{\mathrm{RISC}}=\frac{2\pi}{\hbar\sqrt{4\pi\lambda k_{B}T}}\left| \left\langle\psi_{S} | \hat{H}_{\mathrm{SOC}} | \psi_{T} \right\rangle\right|^{2}\exp\left( -\frac{E_{a}^{RISC}}{k_{B}T} \right)\#\left( S2.3 \right) \end{aligned}$$

where $\left\langle\psi_{S} | \hat{H}_{\mathrm{SOC}} | \psi_{T} \right\rangle$ is the spin-orbit coupling matrix element (SOCME). It can be written for the Arrhenius plot ($\ln k$ vs $1/T$) as Eq. S2.4.

$$\begin{aligned} \ln k_{\mathrm{RISC}}=-\frac{E_{a}^{RISC}}{k_{B}}\cdot\frac{1}{T}+\ln\left( \frac{2\pi}{\hbar\sqrt{4\pi\lambda k_{B}T}}\left| \left\langle\psi_{S} | \hat{H}_{\mathrm{SOC}} | \psi_{T} \right\rangle\right|^{2} \right)\#\left( S2.4 \right) \end{aligned}$$

In this case, there is time dependence term in the intercept coefficient. The phenomena related to TADF provided in very small energy region, this would affect a large impact. Therefore, the Marcus plot with Eq. S2.5 should be used to discuss its temperature dependency (Fukuzumi et al., 2015).

$$\begin{aligned} \ln\left( \sqrt{T}k_{\mathrm{RISC}} \right)=-\frac{E_{a}^{RISC}}{k_{B}}\cdot\frac{1}{T}+\ln\left( \frac{2\pi}{\hbar\sqrt{4\pi\lambda k_{B}}}\left| \left\langle\psi_{S} | \hat{H}_{\mathrm{SOC}} | \psi_{T} \right\rangle\right|^{2} \right)\#\left( S2.5 \right) \end{aligned}$$

From the slope coefficient $\alpha$ of the straight-line approximation in the Marcus plot, $E_{a}^{RISC}$can be explained as $-\alpha k_{B}.$ From the intercept coefficient $\ln\beta$ of the collinear approximation line in the Marcus plot, The absolute value of SOCME can be obtained as Eq. S2.6.

$$\begin{aligned} \left| \left\langle\psi_{S} | \hat{H}_{\mathrm{SOC}} | \psi_{T} \right\rangle\right|=\sqrt{\frac{\hbar}{2\pi}\sqrt{4\pi\lambda k_{B}}\exp(\ln\beta)}\#\left( S2.6 \right) \end{aligned}$$

Because $\Delta G^{\ddagger}$ and $\Delta G^{o}$ are corresponding to be activation energy ($E_{a}^{RISC}$) and energy difference between S_1_ and T_1_ ($\Delta E_{\mathrm{ST}}$), $\lambda$ can be written as Eq. S2.7. Note that $\Delta E_{\mathrm{ST}}$ is a value defined by $E_{a}^{RISC}-E_{a}^{ISC}$ and $\lambda$ is defined as a larger or smaller value than $\Delta E_{\mathrm{ST}}$ in Marcus’ normal or reverse region, respectively.

$$\begin{aligned} \lambda=2E_{a}^{RISC}-\Delta E_{\mathrm{ST}}\pm2\sqrt{E_{a}^{RISC}\left( E_{a}^{RISC}-\Delta E_{\mathrm{ST}} \right)}\#\left( S2.7 \right) \end{aligned}$$

Note that the value of $\Delta E_{\mathrm{ST}}$ should be negative by using $E_{a}^{ISC}$ instead of $E_{a}^{RISC}$. The value of $\lambda$ should be the same between ISC and RISC processes considering the Marcus’s parabolas.

# Derivation of internal quantum efficiency ($\boldsymbol{\eta}_{\boldsymbol{int}}$).

Because the excitons are statistically generated as singlet and triplet with the ratio of 1:3, the prompt emission fraction in current excitation is $0.25\Phi_{PF}$. When the number of cycles for ISC/RISC (*n*) equals 1, the additional emissive fraction is $0.25\Phi_{PF}\Phi_{ISC}\Phi_{RISC}+0.75\Phi_{PF}\Phi_{RISC}$. When *n* = 2 and 3, the additional emissive fraction is $0.25\Phi_{PF}\left( \Phi_{ISC}\Phi_{RISC} \right)^{2}+0.75\Phi_{PF}\Phi_{ISC}{\Phi_{RISC}}^{2}$ and $0.25\Phi_{PF}\left( \Phi_{ISC}\Phi_{RISC} \right)^{3}+0.75\Phi_{PF}{\Phi_{ISC}}^{2}{\Phi_{RISC}}^{3}$, respectively. Therefore, the total emission efficiency considering PF and DF at the current excitation (internal quantum efficiency, $\eta_{int}$) can be formulated as Eq. S3.1 under the assumption of no phosphorescence in emission.

$$\begin{aligned} \eta_{int}&=\frac{1}{4}\Phi_{PF}\sum_{n=0}^{\infty} \left( \Phi_{ISC}\Phi_{RISC} \right)^{n}+\frac{3}{4}\Phi_{PF}\sum_{n=1}^{\infty} {\Phi_{ISC}}^{n-1}{\Phi_{RISC}}^{n} \\ &=\frac{1}{4}\Phi_{PF}+\frac{\left( \Phi_{ISC}+3 \right)}{4\Phi_{ISC}}\Phi_{PF}\sum_{n=1}^{\infty} \left( \Phi_{ISC}\Phi_{RISC} \right)^{n}\#\left( S3.1 \right) \end{aligned}$$

On the other hand, the photoluminescence quantum yield ($\Phi_{PLQY}$) can be written by,

$$\begin{aligned} \Phi_{PLQY}&=\Phi_{PF}\sum_{n=0}^{\infty} \left( \Phi_{ISC}\Phi_{RISC} \right)^{n} \\ &=\Phi_{PF}+\Phi_{PF}\sum_{n=1}^{\infty} \left( \Phi_{ISC}\Phi_{RISC} \right)^{n}\#\left( S3.2 \right) \end{aligned}$$

From Eq. S3.2, the fragment of delayed fluorescence can be written by,

$$\begin{aligned} \Phi_{DF}=\Phi_{PLQY}-\Phi_{PF}=\Phi_{PF}\sum_{n=1}^{\infty} \left( \Phi_{ISC}\Phi_{RISC} \right)^{n}\#\left( S3.3 \right) \end{aligned}$$

From Eq. S3.1 and S3.3, $\eta_{int}$ can be written as

$$\begin{aligned} \eta_{int}&=\frac{1}{4}\Phi_{PF}+\frac{\left( \Phi_{ISC}+3 \right)}{4\Phi_{ISC}}\Phi_{DF}& \\ &=\frac{1}{4}\left( \Phi_{PLQY}+3\frac{\Phi_{DF}}{\Phi_{ISC}} \right)\#\left( S3.4 \right) \end{aligned}$$

Also, $\eta_{int}={\Phi_{PLQY}\left( 1+3\Phi_{RISC} \right)}/4$ can be obtained by a similar method. When the limit condition of $k_{nr}^{T}=0$ was employed, ${\Phi_{DF}}/{\Phi_{ISC}}$ should be $\Phi_{PLQY}$ because $\Phi_{DF}$ is the emitting fraction via S_1_ of generated T_1_ exciton by ISC process. Therefore, $\eta_{int}$ is provided as the same value with $\Phi_{PLQY}$. However, $\eta_{int}$ is provided much smaller than $\Phi_{PLQY}$ in the limit condition of $k_{nr}^{S}=0$. In the case of 1 wt% **BN4** in DPEPO, $\eta_{int}$ was estimated to be 0.801 (= $\Phi_{PLQY}$) and 0.459 for $k_{nr}^{T}=0$ and $k_{nr}^{S}=0$, respectively.

# Supplementary Figures and Tables.

**
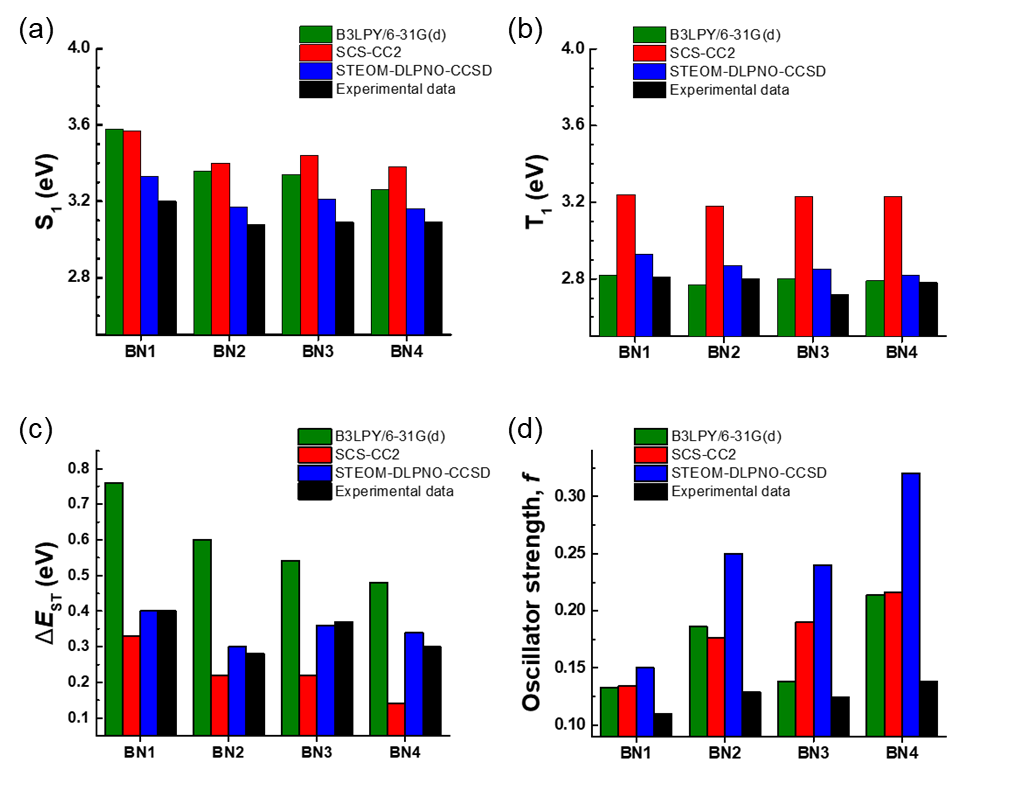
Figure S1**. Comparison of various calculation results and experimental data (in toluene) of **BN1**-**4** for (a) S_1_ energies, (b) T_1_ energies, (c) Δ*E*_ST_, and (d) oscillator strength (*f*).

**Table S1.** Calculated excitation energies and oscillator strength with various levels of theories and experimental results. Spin orbit-coupling value was also calculated with the STEOM-DLPNO-CCSD method.

|  | **B3LPY/6-31G(d)** | | | |  | **SCS-CC2** | | | |  | **STEOM-DLPNO-CCSD** | | | | | **Experimental (in toluene) ^[a,c]^** | | | | | **Experimental (in DPEPO) ^[b,c]^** | | | | |
| --- | --- | --- | --- | --- | --- | --- | --- | --- | --- | --- | --- | --- | --- | --- | --- | --- | --- | --- | --- | --- | --- | --- | --- | --- | --- |
|  | **S_1_ (eV)** | **T_1_ (eV)** | **Δ*E*_ST_ (eV)** | ***f*** |  | **S_1_ (eV)** | **T_1_ (eV)** | **Δ*E*_ST_ (eV)** | ***f*** |  | **S_1_ (eV)** | **T_1_ (eV)** | **Δ*E*_ST_ (eV)** | ***f*** | **SOC (cm^-1^)** |  | **S_1_ (eV)** | **T_1_ (eV)** | **Δ*E*_ST_ (eV)** | ***f* ^[d]^** | |  | **S_1_ (eV)** | **T_1_ (eV)** | **Δ*E*_ST_ (eV)** |
| **BN1** | 3.58 | 2.82 | 0.76 | 0.133 |  | 3.57 | 3.24 | 0.33 | 0.134 |  | 3.33 | 2.93 | 0.40 | 0.15 | 0.00 |  | 3.20 | 2.81 | 0.40 | 0.110 | |  | 3.18 | 2.82 | 0.36 |
| **BN2** | 3.36 | 2.77 | 0.60 | 0.186 |  | 3.40 | 3.18 | 0.22 | 0.176 |  | 3.17 | 2.87 | 0.30 | 0.25 | 0.00 |  | 3.08 | 2.80 | 0.28 | 0.129 | |  | 3.06 | 2.78 | 0.28 |
| **BN3** | 3.34 | 2.80 | 0.54 | 0.138 |  | 3.44 | 3.23 | 0.22 | 0.190 |  | 3.21 | 2.85 | 0.36 | 0.24 | 0.19 |  | 3.09 | 2.72 | 0.37 | 0.124 | |  | 3.07 | 2.76 | 0.29 |
| **BN4** | 3.26 | 2.79 | 0.48 | 0.214 |  | 3.38 | 3.23 | 0.14 | 0.216 |  | 3.16 | 2.82 | 0.34 | 0.32 | 0.12 |  | 3.09 | 2.78 | 0.30 | 0.138 | |  | 3.07 | 2.83 | 0.24 |

[a] 1.0 × 10^-5^ mol L^-1^. [b] Doping concentration of 1 wt%. [c] Onset value. [d] Estimated from absorption and emission spectra by reported method in literature (Tsuchiya et al., 2020).


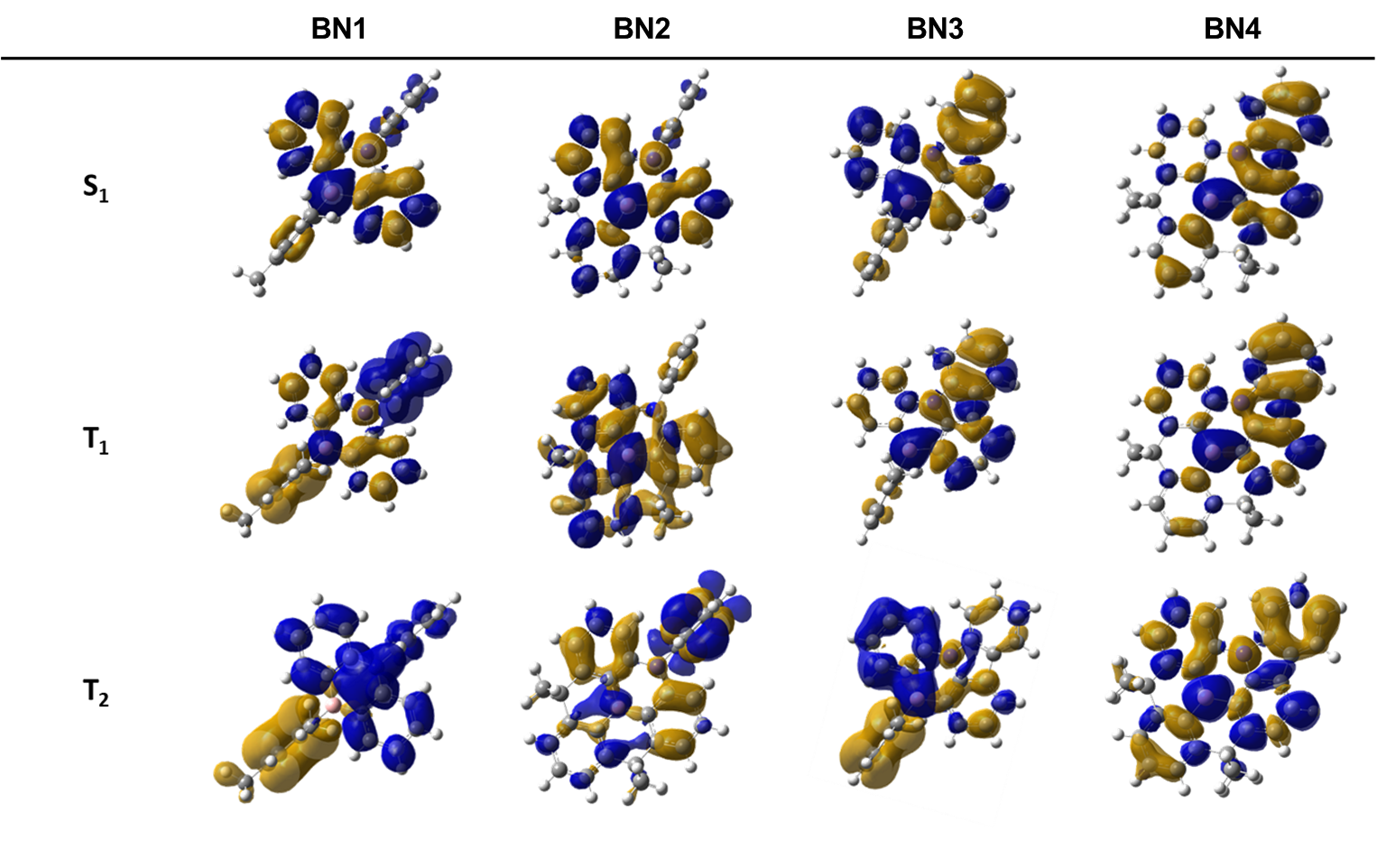


**Figure S2.** Spin density distribution plots of **BN1**, **2**, **3**, and **4** for optimized structures on each state at STEOM-DLPNO-CCSD level of theory. Distributions indicating blue and yellow denote electron-withdrawing and donating characteristics, respectively.


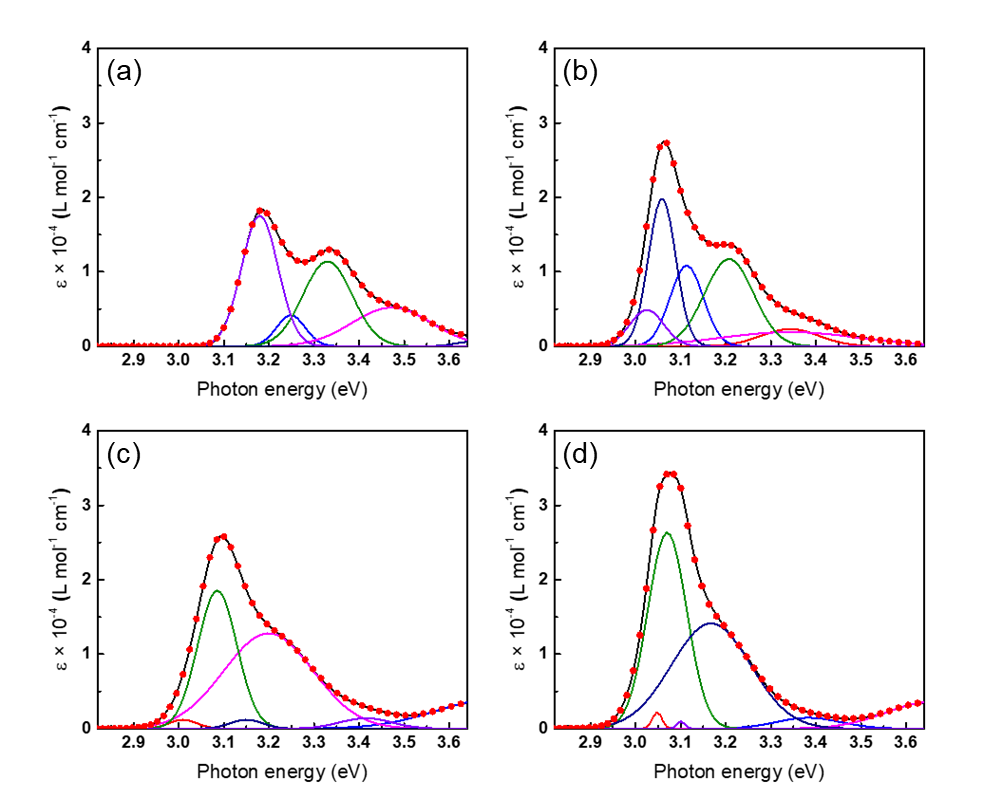


**Figure S3**. Absorbance characteristics of BN molecules in toluene solution; (a) **BN1**, (b) **BN2**, (c), **BN3**, and (d) **BN4**. Black lines are experimental data from UV-vis measurement. Colored lines represent fitting curves based on the Gaussian model: ε_n_(ν) = A_n_ exp(−(ν−B_n_)^2^/2C_n_^2^) (n = 1, 2, 3…), where A_n_ is the amplitude, B_n_ is the average wavenumber and Cn is the distribution parameter. Dotted red lines are a summation of each fitting curve. Since the **BN**s showed strong absorption spectra by short-range charge transfer between HOMO-LUMO, the summation of the Gaussian model fitting curves constituting the first absorption spectrum was used to obtain *f*, *Q*, and *k*_r_^S^ values. Estimation method was followed within the literature (Tsuchiya et al., 2020).


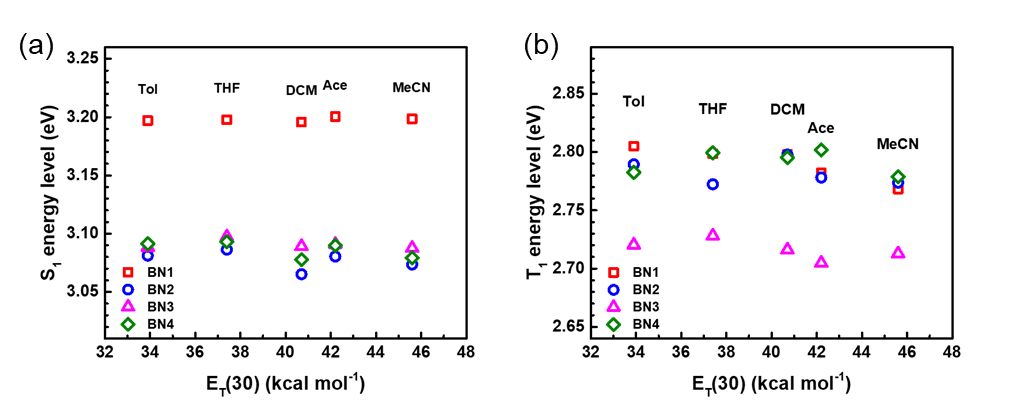


**Figure S4**. S_1_ (a) and T_1_ (b) energy levels of **BN**s in various solvents in *E*_T_(30) scales as a solvent polarity parameter (Reichardt et al., 1994). S_1_ and T_1_ energy levels are estimated from onset values of fluorescence and phosphorescence spectra, respectively.


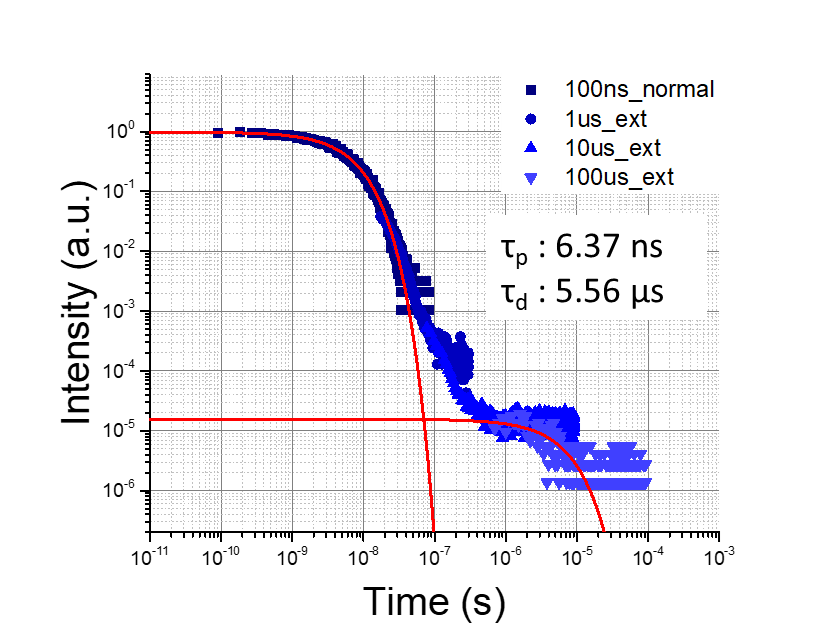


**Figure S5**. Emission decay of **BN4** in toluene solution measured by dynamic-range streak camera system; the difference from theoretical curves and observed plots are based on the IRF.


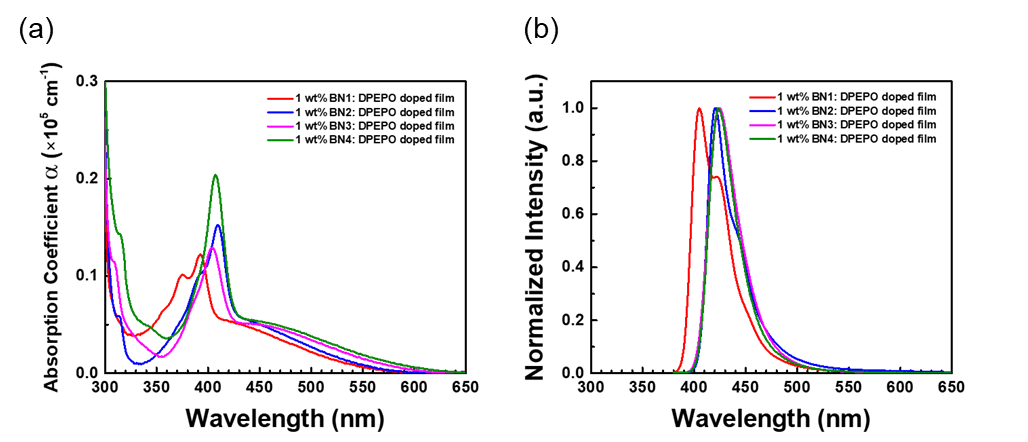


**Figure S6**. (a) Absorption and (b) fluorescence spectra of 1 wt% **BN**s in DPEPO film.


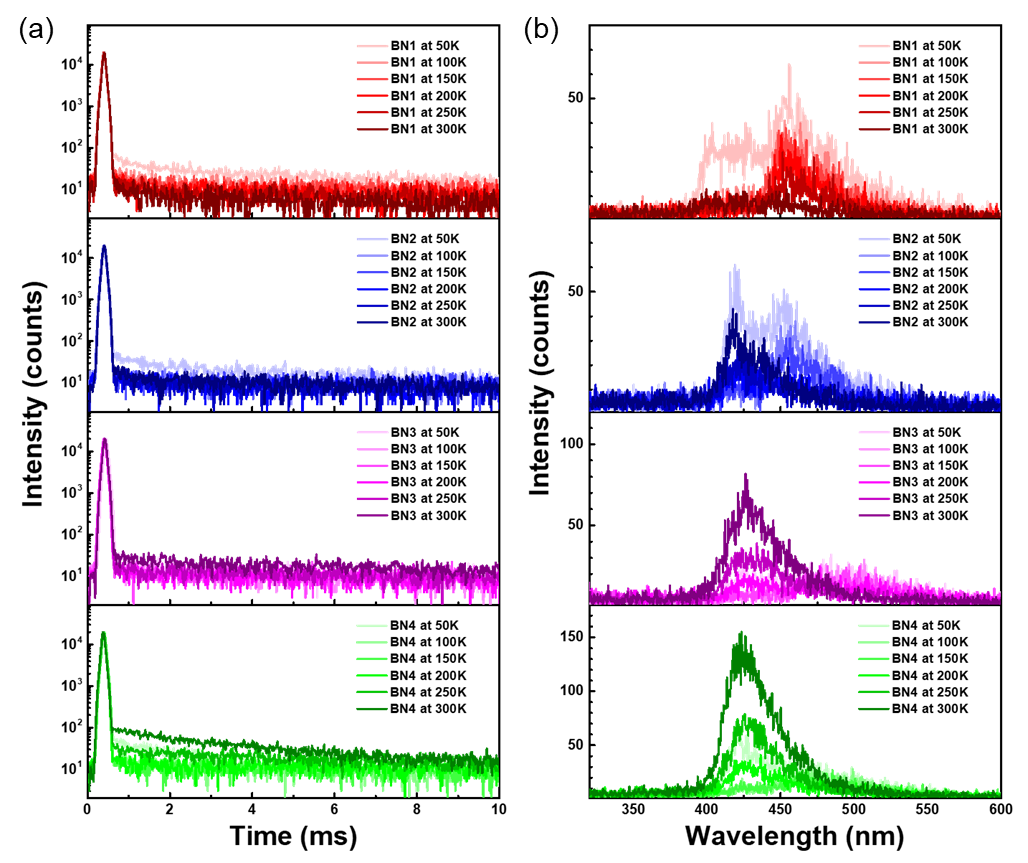


**Figure S7.** (a) Temperature dependency of transient photoluminescence decay curves of **BN**s in DPEPO films. (b) Transient emission spectra for delayed emission of 1-10 ms time range.

**Table S2.** Parameters for RISC process estimated by Marcus equation.

|  | **BN1** | **BN2** | **BN3** | **BN4** |
| --- | --- | --- | --- | --- |
| ${\Delta E}_{S_{1}-T_{1}}$ (eV) ^[a]^ | 0.40 | 0.30 | 0.36 | 0.34 |
| $\mathrm{SOCME}_{S_{1}-T_{1}}$ (cm^-1^) ^[a]^ | 0.00 | 0.00 | 0.19 | 0.12 |
| $k_{RISC}^{S_{1}\leftarrow T_{1}}$ (s^-1^) ^[a,b]^ | − | − | 1.39×10^1^ | 1.23×10^1^ |
| ${\Delta E}_{S_{1}-T_{2}}$ (eV) ^[a]^ | 0.12 | 0.02 | -0.10 | 0.23 |
| $\mathrm{SOCME}_{S_{1}-T_{2}}$ (cm^-1^) ^[a]^ | 0.01 | 0.04 | 0.10 | 0.08 |
| $k_{RISC}^{S_{1}\leftarrow T_{2}}$ (s^-1^) ^[a,b]^ | 7.16×10^2^ | 1.34×10^6^ | 8.14×10^6^ | 4.70×10^2^ |
| ${\Delta E}_{T_{1}-T_{2}}$ (eV) ^[a]^ | 0.28 | 0.28 | 0.46 | 0.11 |
| Boltzmann factor ^[a]^ | 1.98×10^-5^ | 1.98×10^-5^ | 1.87×10^-8^ | 1.42×10^-2^ |
| $k_{RISC}^{S_{1}\leftarrow T_{2}\leftrightarrow T_{1}}$ (s^-1^) ^[a,b]^ | 1.42×10^-2^ | 2.66×10^1^ | 1.40×10^1^ | 1.88×10^1^ |
| $k_{RISC}^{nrS=0}$ (s^-1^) ^[c]^ | − | 1.68×10^1^ | 2.93×10^1^ | 1.19×10^2^ |
| ${\Delta E}_{\mathrm{ST}}$ (eV) ^[d]^ | 0.36 | 0.28 | 0.29 | 0.24 |
| $\mathrm{SOCME}_{S_{1}-T_{1}}$ (cm^-1^) ^[b,c,d]^ | − | 0.042 | 0.068 | 0.049 |
| ${\Delta E}_{\mathrm{ST}}$ (eV) ^[e]^ | − | − | 0.065 | 0.121 |
| λ (eV) ^[e]^ | − | − | 0.065 | 0.121 |
| $\mathrm{SOCME}_{S_{1}-T_{1}}^{\mathrm{effective}}$ (cm^-1^) ^[e]^ | − | − | 0.00059 | 0.00398 |
| $k_{RISC}^{nrT=0}$ (s^-1^) ^[c]^ | − | 6.68×10^1^ | 8.53×10^1^ | 2.75×10^2^ |
| ${\Delta E}_{\mathrm{ST}}$ (eV) ^[d]^ | 0.36 | 0.28 | 0.29 | 0.24 |
| $\mathrm{SOCME}_{S_{1}-T_{1}}$ (cm^-1^) ^[b,c,d]^ | − | 0.083 | 0.115 | 0.075 |
| ${\Delta E}_{\mathrm{ST}}$ (eV) ^e)^ | − | − | 0.099 | 0.083 |
| λ (eV) ^[e]^ | − | − | 0.120 | 0.184 |
| $\mathrm{SOCME}_{S_{1}-T_{1}}^{\mathrm{effective}}$ (cm^-1^) ^[e]^ | − | − | 0.00065 | 0.00435 |

[a] Theoretical calculation values (Samanta et al. 2017). [b] ${\Delta E}_{S_{1}-T_{x}}$ employing as an activation energy. [c] Experimentally obtained emission decay at 300 K. [d] Fluorescence and phosphorescence spectra. [e] Marcus plot assuming in normal region.


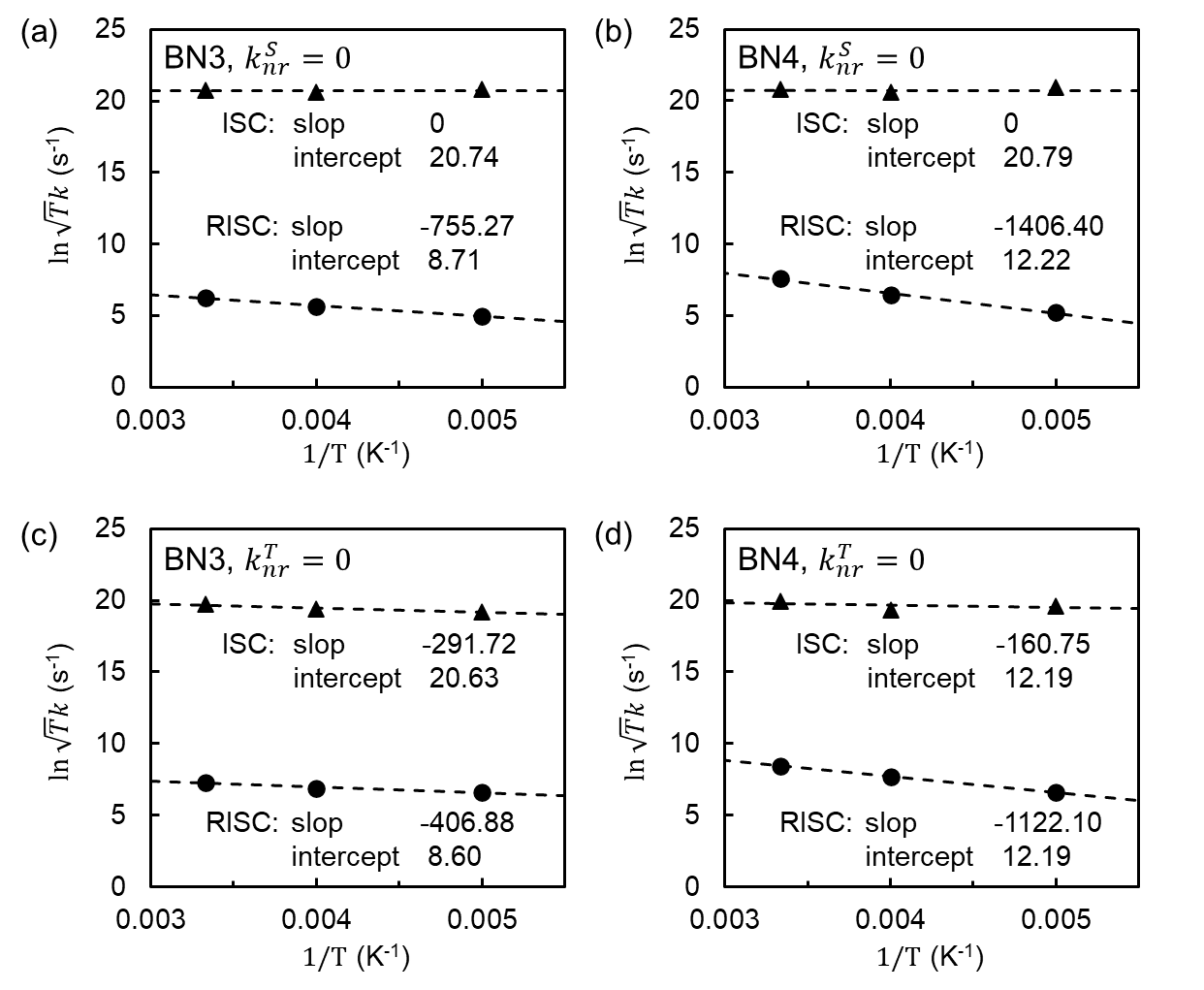


**Figure S8.** Marcus plots (ISC and RISC rate constants vs inverse of temperature) for **BN3** (a, c) and **BN4** (b, d) in case of limit condition of $k_{nr}^{S}=0$ (a, b) and $k_{nr}^{T}=0$ (c, d).

**References**

Fukuzumi, S., Itoh, A., Ohkubo, K., and Suenobu, T. (2015). Size-selective incorporation of donor–acceptor linked dyad cations into zeolite Y and long-lived charge separation. *RSC Adv.*, 5, 45582−45585. doi: 10.1039/c5ra06165b.

Marcus, R. A. (1993). Electron transfer reactions in chemistry. Theory and experiment”, *Rev. Mod. Phys.*, 65, 599−610.

Reichardt, C. (1994). Solvatochromic dyes as solvent polarity indicators. *Chem. Rev.* 94, 2319–2358. doi: 10.1021/cr00032a005.

Samanta, P. K., Kim, D., Coropceanu, V., and Brédas, J.-L. (2017). Up-Conversion Intersystem Crossing Rates in Organic Emitters for Thermally Activated Delayed Fluorescence: Impact of the Nature of Singlet vs Triplet Excited States. *J*. *Am*. *Chem*. *Soc*., 139, 4042−4051. doi: 10.1021/jacs.6b12124.

Tsuchiya, Y., Tsuji, K., Inada, K., Bencheikh, F., Geng, Y., Kwak, H. S., et al. (2020). Molecular Design Based on Donor-Weak Donor Scaffold for Blue Thermally-Activated Delayed Fluorescence Designed by Combinatorial DFT Calculations. *Front. Chem.* 8, 2–11. doi: 10.3389/fchem.2020.00403.
